# Increase in the extent of mass coral bleaching over the past half-century, based on an updated global database

**DOI:** 10.1371/journal.pone.0281719

**Published:** 2023-02-13

**Authors:** Alejandra Virgen-Urcelay, Simon D. Donner

**Affiliations:** 1 Institute for Resources, Environment and Sustainability, University of British Columbia, Vancouver, British Columbia, Canada; 2 Department of Geography, University of British Columbia, Vancouver, British Columbia, Canada; Newcastle University, UNITED KINGDOM

## Abstract

The recurrence of mass coral bleaching and associated coral mortality in the past few decades have raised questions about the future of coral reef ecosystems. Although coral bleaching is well studied, our understanding of the spatial extent of bleaching events continues to be limited by geographical biases in data collection. To address this gap, we updated a previous observational database and spatially modelled the probability of past bleaching occurrence. First, an existing raw observational database was updated to cover the 1963–2017 period using searches of the academic and grey literature and outreach to coral reef monitoring organizations. Then, in order to provide spatially-explicit global coverage, we employed indicator kriging to spatially model the probability of bleaching occurrence each year from 1985 through 2017 at 0.05° x 0.05° lat-long resolution. The updated raw database has 37,774 observations, including 22,650 positive bleaching reports, three times that in the previous version. The spatial interpolation suggests that 71% of the world’s coral reefs likely (>66% probability) experienced bleaching at least once during the 1985 and 2017 period. The mean probability of bleaching across all reefs globally was 29–45% in the most severe bleaching years of 1998, 2005, 2010 and 2016. Modelled bleaching probabilities were positively related with annual maximum Degree Heating Weeks (DHW), a measure of thermal stress, across all years (p<0.001), and in each global bleaching event (p<0.01). In addition, the annual maximum DHW of reef cells that very likely (>90% probability) experienced bleaching increased over time at three times the rate of all reef cells, suggesting a possible increase in reef thermal tolerance. The raw and spatially interpolated databases can be used by other researchers to enhance real-time predictions, calibrate models for future projections, and assess the change in coral reef response to thermal stress over time.

## Introduction

Exposure to anomalous environmental conditions can cause coral bleaching; a paling of warm-water corals and other reef species due to the breakdown of the symbiosis between the host animals and the dinoflagellate algae residing within their tissue [1]. A variety of environmental stressors including low salinity, sedimentation, aerial exposure, and pollutants can cause coral bleaching, but for reef-scale or mass coral bleaching events, prolonged temperature extremes are the most common driver [2]. The increase in mass coral bleaching and associated loss of live coral cover due to more frequent, extreme and expansive marine heatwaves (MHWs) have raised concerns about the future of coral reefs in a warming ocean [3, 4]. Over last three decades, there have been global-scale coral bleaching events in 1997–1998, 2009–2010 and 2014–2017 [4–6] as well as many regional events during the intervening years [7–8]. Projected increases in sea surface temperature (SST) over the next few decades are expected to further increase the frequency of the conditions that can cause mass coral bleaching [9–12] and contribute to a decline in living coral cover and reef carbonate production [13, 14].

Accurate and comprehensive historical coral bleaching observations are critical for informing real-time prediction of coral reef response to climate extremes [15], detecting evidence for acclimation or ‘ecological memory’ [16] and projecting the response of coral reefs to future warming and MHWs [13]. Though global databases of bleaching observations have been compiled [4, 17], they are limited by the decentralized nature of coral reef monitoring and unequal sampling effort across reef locations [8]. Some bleaching events can go unnoticed in reefs due to their remote location [18]. Additionally, there is an uneven global distribution of bleaching reports due to geographical biases in the surveying effort [8]. For instance, the reports compiled in the widely known online database ReefBase (reefbase.org) are mostly clustered in developed countries and areas of high research interest, like the Caribbean, the Great Barrier Reef and Florida.

A previous effort from our group [8] attempted to address those limitations by developing the first gridded, global-scale historical coral bleaching database that offers annual global maps of the probability of bleaching occurrence. The publicly-available database includes raw observational data for 1963 through 2010 and spatially-interpolated bleaching probabilities for 1985 through 2010, thus providing insights into the extent of bleaching into some of the most extensive global-scale events (e.g., 1997–1998, 2009–2010). Though it represented an advance on previous voluntary, coarse or regional datasets, the raw observational database still lacked sufficient coverage to enable interpolation in many years and regions. The database also concluded before the 2014–2016 global-scale bleaching event, the most extensive in recorded history [7]. An updated version of the database is needed to better serve the initial goal of tracking the extent of bleaching over time, as well as to enhance real-time prediction, test hypotheses about acclimation or adaptation, and examine response to multiple drivers. A recent data manuscript [17] began to address these latter goals by combining observations from existing databases, including [8], and coral reef monitoring organizations with key environmental variables in one dataset. Unfortunately, since coral reef monitoring and reporting of bleaching events are still decentralized, further mining of the literature and outreach to managers and researchers in regions underrepresented in the previous datasets and monitoring programs is necessary to achieve the global coverage necessary to spatially reconstruct the change in bleaching extent over time.

In this paper, we describe the development of updated global-scale historical bleaching databases and use those databases to conduct a preliminary assessment of the evolving relationship between thermal stress and coral bleaching. First, we describe the development of a new observational database (hereafter termed ‘version 2’) containing 37,774 reports from 1963–2017, including a three-fold increase in positive bleaching observations over the original database (‘version 1’). Second, we use the raw observational database and an updated indicator kriging procedure to estimate annual bleaching probability for all 0.05° x 0.05° coral reef cells from 1985 through 2017. Finally, to demonstrate potential applications of the database, we use the annual bleaching probability data to assess the extent of major global bleaching events (1997–1998, 2009–2010 and 2014–2016) and to test the hypothesis that the likelihood of bleaching increases with thermal stress, at regional and global scales.

## Materials and methods

### Observational data

The raw observational database follows the format and protocol used in version 1 [8], which includes categories for source, country, site names, latitude and longitude, year, month, percent bleached, bleaching severity code, percent mortality, mortality severity code, depth, and survey method (S1 Table). The categorical severity code for bleaching and mortality variables (where available) follow the same protocol used in Reefbase: with 0 = no bleaching (<1%), 1 = mild bleaching (1–10% bleached), 2 = moderate (10–50% bleached), and 3 = severe (>50% bleached). To be included in the database, each report must at minimum include latitude, longitude, year, bleaching severity code and source; the categorical variables are necessary in addition to the continuous percent variables because many early reports roughly estimate the fraction of living corals that bleached or died.

The database was updated through a targeted search for observations of bleaching in the academic and grey literature (using Google Scholar and searches of all International Coral Reef Symposium archives) and in the voluntary online database Reefbase. We also contacted scientists who reported bleaching to the international Coral-List (https://coral.aoml.noaa.gov/mailman/listinfo/coral-list/) as well as representatives of coral reef monitoring organizations for bleaching observations. The organizations which contributed observations include those whose data also appears in [17] (e.g., Atlantic and Gulf Rapid Reef Assessment, ReefCheck, Florida Reef Resilience Program, Wildlife Conservation Service) and others which conducted surveys in underrepresented regions (e.g., Coral Cay Conservation, XL Catlin Seaview Survey). This search aimed to infill gaps in the pre-2010 data from the previous version of the database and to update the database through the year 2017. NOAA Coral Reef Watch also contributed its internal database of reports collected as part of research on 2014–2017 global coral bleaching event. In addition, in order to improve usability of the database, we also reviewed each report from [8] which either lacked the percent bleached, lacked the month or day of report, or reporting thermal bleaching during the cold season, and infilled or corrected the information based on the original source material where possible.

All new and previous reports were submitted to a new quality control procedure to correct errors in the coordinate of observations, which are common in source data because of different conventions in reporting coordinates, evolving accuracy of GPS devices and the proximity of reefs to land. First, to identify inaccurate coordinates, we used ArcMap version 10.6.1 and Google Earth to verify that the coordinates corresponded with the country and/or region listed for each report. Coordinates were manually corrected where possible using other location information. In the cases where the coordinates were positioned on land, alternative coordinates corresponding to the closest coral reef location were estimated by obtaining the shortest distance between each report and a coral reef location using the distance matrix tool from QGIS software version 3.8. Coral reef locations were defined with a 0.05° x 0.05° raster layer developed using the Millennium Coral Reef Mapping Project dataset [19] as a reference, with cells added to correspond with the coordinates of correct observed bleaching reports not labelled as reefs in [19].

### Spatial interpolation

Annual 0.05° x 0.05° lat-long resolution global maps of probability of bleaching occurrence for a given year (between 1985 and 2017) were developed using indicator kriging, a geostatistics technique that estimates of unknown values by assigning weights to each of the sample points that are proximate to the estimate [20]. Unlike ordinary and other forms of kriging, indicator kriging employs binary indicator data to estimate the probability of exceeding a threshold value (e.g., pollutant exceeding a threshold) or belonging to a particular class (e.g., species is present). This method is commonly used to create spatial maps from presence or absence data, as in species distribution models [21–23], mineral resource estimation, and other natural resource mapping applications [24]. Predictions from indicator kriging are based on the model:

$$I(s)=\mu +\varepsilon^{\prime }(s)$$

Where *I*(*s*) is a binary variable, *μ* represents an unknown constant (global mean) and *ε*′(*s*) is the spatially correlated stochastic component of variation [25]. The locations for which a value is being estimated (in this case, each grid cell with coral reefs) are surrounded by actual sampling points (observational bleaching data) and are within the spatial auto-correlation range [25]. A semivariogram is developed to estimate the degree of variance between multiple pairs of sample points separated by a distance and provide information on the scale and pattern of spatial variance [26]. By fitting a mathematical model to the semivariance estimates, indicator kriging can be used to estimate the probability of presence (a value from 0 to 1) for all locations [24].

Here, indicator kriging was conducted for each year from 1985 through 2017 using the R statistical computing environment and RStudio software. The analysis was conducted independently for each of four ocean regions each year (Caribbean, Indian Ocean, Pacific Ocean, East Pacific; see S1 Fig) and then combined into a global map, in order to reduce computational demand while still preserving spatial autocorrelation within each basin. In each year, bleaching presence was defined as any 0.05° x 0.05° lat-long grid cell that contained a report with severity code >1. The mild (<10% bleaching) reports were excluded from the indicator kriging because they are most likely to represent mistaken observations, non-thermal events (e.g., low tides, freshwater flux) or minor non-lethal shallow summer events, previously found to be common problems in voluntary monitoring programs [8, 27].

Bleaching pseudo-absence was defined by a lack of thermal stress, based on the assumption that heat-driven bleaching was unlikely with no or low thermal stress. Available absence data (i.e. severity code 0 in the raw observational database) is too limited due to the ‘streetlight effect’: literature and monitoring focusing disproportionately on occurrences of bleaching, whereas no bleaching is the far more common state for reefs. In version 1 [8], pseudo-absences were defined each year as grid cells in which the Degree Heating Week (DHW) value, a thermal stress metric based on the accumulation of anomalous SST over the previous 12 weeks [15], was zero all year. However, since the day-to-day variability in satellite-derived SSTs can lead to non-zero but low (<1°Cˑweek) DHW values occurring even during years with no significant accumulation of thermal stress, a pseudo-absence criteria of DHW = 0°Cˑweek may omit many instances where thermal bleaching is highly unlikely to have occurred. Here, we determined the criteria for defining pseudo-absences through a series of sensitivity tests in which the kriging was repeated using four different thresholds (0°Cˑweek, <0.25°Cˑweek, <0.5°Cˑweek, and <1°Cˑweek) for each of the four kriging regions. All analysis was conducted with the DHW data from 0.05° x 0.05° CoralTemp V1 product [28]. In each region, tests were conducted using data from a year with a high abundance of bleaching reports and a year with a low to moderate abundance of bleaching reports (S2 Table). The pseudo-absence threshold of DHW<0.5°Cˑweek was then chosen for this study based on the best fit between interpolated bleaching probabilities and raw bleaching observations across all iterations (S2 Table). With a higher threshold (e.g., DHW<1°Cˑweek), the mean bleaching probability was too low in grid cells with observed reports, whereas with a lower threshold (e.g., DHW = 0°Cˑweek), the semi-variogram failed to converge in some regions and years.

For each combination of kriging and region and year, semivariograms were automatically fitted using autofitVariogram function from the Automap package [29]. This function iterates over eight different mathematical models (Exponential, Spherical, Gaussian, Matern, Stein’s Matern, Circular, Linear, Bessel, and Pentaspherical) and selects the one with the smallest residual sum of squares. The variogram model in the krige function from gstat package [30] using the formula z~1 for ordinary kriging was used. Automatic model selection brings risks of overfitting, which can result in maps with sharp gradients from positive observations [31], and of different mathematical models being selected for each time step [32]. Automap was chosen over the manual fitting procedure from version 1 [8] to improve reproducibility, and because the improved spatial coverage in the raw observational dataset reduced the risk of overfitting. In addition, because each bleaching event has a different spatial pattern, the option of different mathematical models for different regions and years is warranted.

Automap was attempted for all regions and years with data (1985–2017). Secondary oversight was conducted in cases where semivariograms failed to converge or produced no estimations. In these cases, initial estimates for range, sill, and nugget values determined from visual inspection [26, 33] were provided to the autofitVariogram function. Interpolations were not possible to complete in all regions and years because of insufficient data available, which could mean either no or limited bleaching occurred that year, bleaching occurred but was insufficiently reporting, or the available reports were too geographically clustered. In years for which kriging was not possible to complete, grid cells with bleaching reports of severity >1 were assigned a probability of 100% and all other cells in the region were assigned a probability of 0%.

### Data analysis

We tested the accuracy of the modeled bleaching probabilities against the raw observational database at the global level and for ten reef regions defined by [34] (see S2 Fig). For each modeled year, Welch two-sample t-tests were conducted within each reef region between bleaching probabilities of all coral reef cells and the bleaching probabilities for the subset of cells that contained observed bleaching reports; this approach was used because the samples have different means, and unequal variances and sample sizes. We also assessed the difference between the area of observed bleaching according to the interpolated and the raw observational databases by comparing the number of cells that had a likely (>66%) and a very likely probability of bleaching (>90%) with the number of cells with raw observations of severity code >1. This analysis was done globally and regionally, and for the three major global bleaching events (1997–1998, 2009–2010, 2014–2016). To compare the results with those of version 1, we conducted Welch two-sample t-tests between the bleaching probabilities for cells that contained at least one observational bleaching report within a given year and region.

To demonstrate potential applications of the interpolated dataset, we tested whether the mean thermal stress was higher in cells with higher bleaching probability. First, we extracted the annual maximum DHW value from CoralTemp V1.0 data for all reef cells in each modelled year; the annual maximum value was used because the interpolated probability dataset is at the annual scale. Welch two-sample t-tests were then used to test for significant differences between the mean DHW value in different bleaching probability subsets (>90%, >66–90%, >50–66%, >33–50%, >10–33%, ≤10%). This was conducted at the global and regional level, and for the three major global bleaching events. Corrections for experiment-wise multiple tests to reduce Type 1 errors (e.g., Bonferroni) were not applied because these are exploratory tests of a pre-established hypothesis [35], and tests involved data from independent years and regions. We also tested whether the thermal stress at which bleaching occurred increased over time by comparing the change in DHW values for cells that likely and very likely experienced bleaching (>66% and >90% bleaching probability) with that of all cells from 1985 through 2017. All the statistical analyses were done using the R statistical computing environment. The package ggplot2 [36] was used to produce all plots. All maps were produced using ArcGIS Pro version 2.6.

## Results

### Observational bleaching data

**Fig 1:**
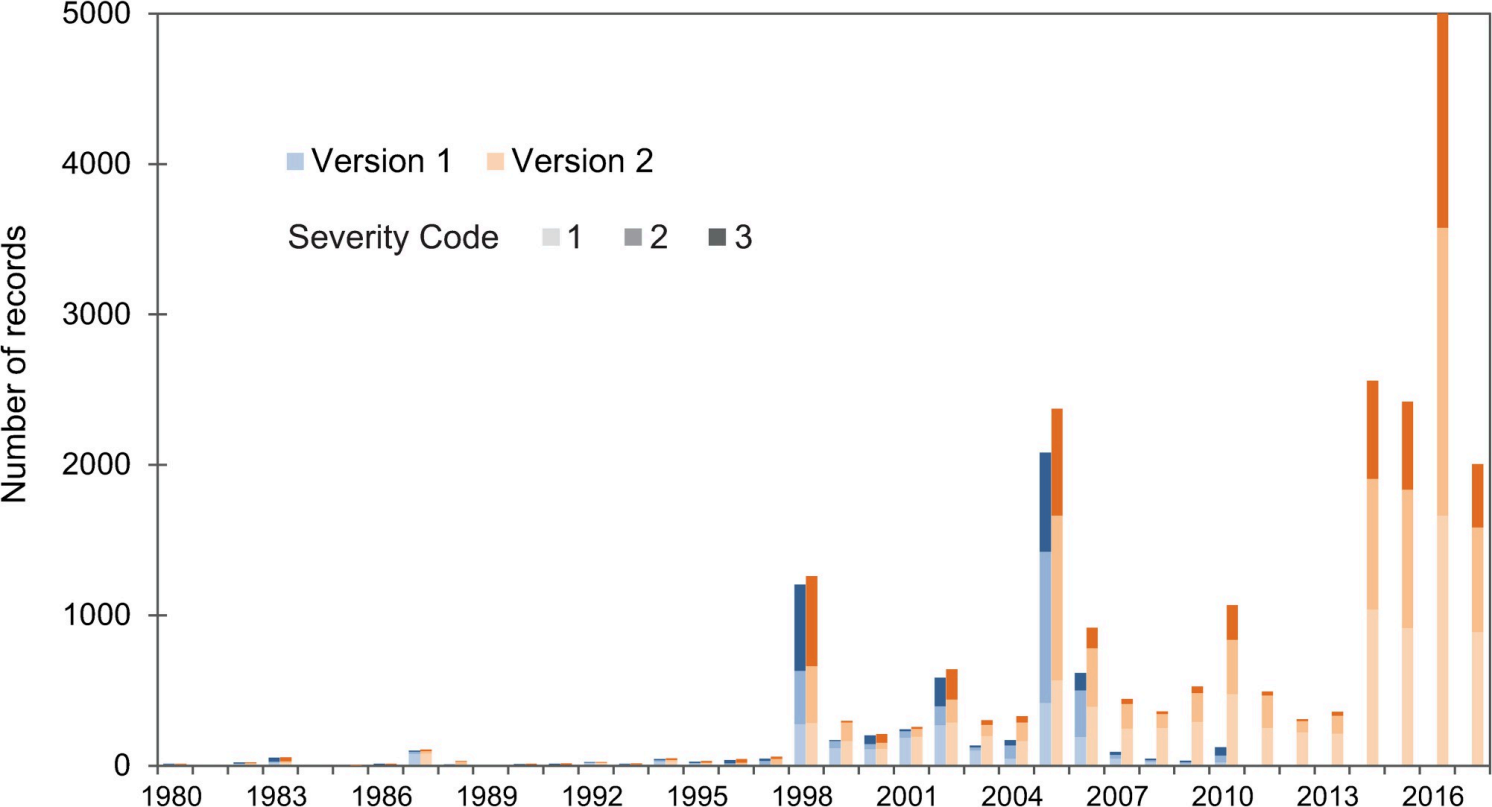
Number of bleaching reports by year for 1985–2017 in version 1 and version 2 of the database. Note that severity code 1 in this figure refers to severity code -1 or 1. Years: 1963, 1969, 1973, 1976–1977, and 1979–1984 do not appear in this figure but had bleaching reports.

The raw observational database shows that bleaching occurred in at least one location in 1963, 1969, 1973, 1976–1977, and each year from 1979 through 2017 (Fig 1). The number of positive bleaching reports increased to 22,650 from 7,437 in version 1; the database also includes 15,124 no bleaching or severity code 0 reports. This is a similar number of total observations (37,774) as the recent compilation of databases by [17] (34,584), but 21% more positive bleaching reports, primarily because [17] includes the full ReefCheck database in which 74% of its 22,325 records classify as no bleaching.

This threefold increase in reports since version 1 is due to additional years of observations (2011–2017) and increased coverage for years prior to 2011, minus the removal of duplicate reports in version 1. Of the positive reports, only 12 (0.1%) occurred before 1980 and only 264 (1.2%) occurred before 1990. The majority of the report (57%) coincided with the four “global” mass coral bleaching events each of which occurred during El Niño events (Table 1). The 1982–1983 event accounts for 0.3% of total reports, whereas the 1997–1998 and 2009–2010 events account for 6% and 7% of total reports, and the 2014–2016 event accounts for 44% of total reports. An additional 10.5% of the reports are from 2005, during which the eastern Caribbean experienced mass bleaching. Most reports from the 1982–1983 event have an unknown severity, whereas reports from the 1997–1998 event were predominantly “severe” (level 3) and reports from 2014–2016 were predominantly “moderate” (level 2).

**Table 1:** Observational bleaching reports for all years and for significant bleaching periods.

Reports		Unknown	Mild	Moderate	Severe	Total
**Version 1**	**1982–1983**	42	0	4	31	77 (1%)
	**1997–1998**	32	354	412	723	1,521 (20%)
	**2009–2010**	93	53	54	109	309 (4%)
	**2014–2016**	-	-	-	-	-
	**All years**	752	2,408	2,046	2,231	7,437
**Version 2**	**1982–1983**	37	0	12	29	78 (0.3%)
	**1997–1998**	32	266	412	609	1,319 (6%)
	**2009–2010**	197	574	551	272	1,594 (7%)
	**2014–2016**	331	3,291	3,702	2,658	9,982 (44%)
	**All years**	1,235	7,896	8,160	5,359	22,650

Because of the geographically uneven sampling effort, the number of bleaching reports can be a misleading measure of the extent of bleaching in a given year or region. Converting point observations to a spatially-explicit grid can reduce bias caused by multiple reports from the same location. The number of 0.05° x 0.05° lat-long grid cells containing positive bleaching reports each year suggests a different ranking of the most severe bleaching years (S3 Fig). The gridded data suggest that 2016 experienced the greatest extent of bleaching; this is followed by 1998, even though 2005, 2014, and 2015 featured more bleaching reports than 1998.

The geographical biases in coral reef monitoring and bleaching reporting is apparent in the regional data. The Caribbean featured the highest number of grid cells with at least one positive bleaching report over time, followed by Australia and SE Asia (S4 Fig). When analysed with respect to the total reef area in each region, however, the East Pacific had the highest proportion of cells with reports (37.3%), followed by the Caribbean (22.3%) and Australia (16.4%) regions, whereas SE Asia had the second lowest proportion of cells with reports (4.7%).

### Interpolated bleaching probabilities

The observational bleaching reports were sufficient to produce interpolated maps of bleaching probabilities for 27 of the 33 years within 1985 through 2017, adding nine years of data (1991, 1994, and 2011–2017) compared to version 1 (S3 Table). Eleven years had sufficient reports to produce interpolated maps for all three of the primary kriging regions (Caribbean, Indian Ocean, Pacific Ocean); only three years (1998, 2015, and 2016) had sufficient reports to interpolate in all three plus the fourth, smaller East Pacific region. In total, interpolation was possible in 60 combinations of regions and years, close to twice the number (31) in version 1 of the database. There was also no statistically significant difference (p<0.05) between the bleaching probabilities of all cells with reports with version 1 of the dataset for 21 of the 29 regions and years modelled in both versions (S4 Table).

**Fig 2:**
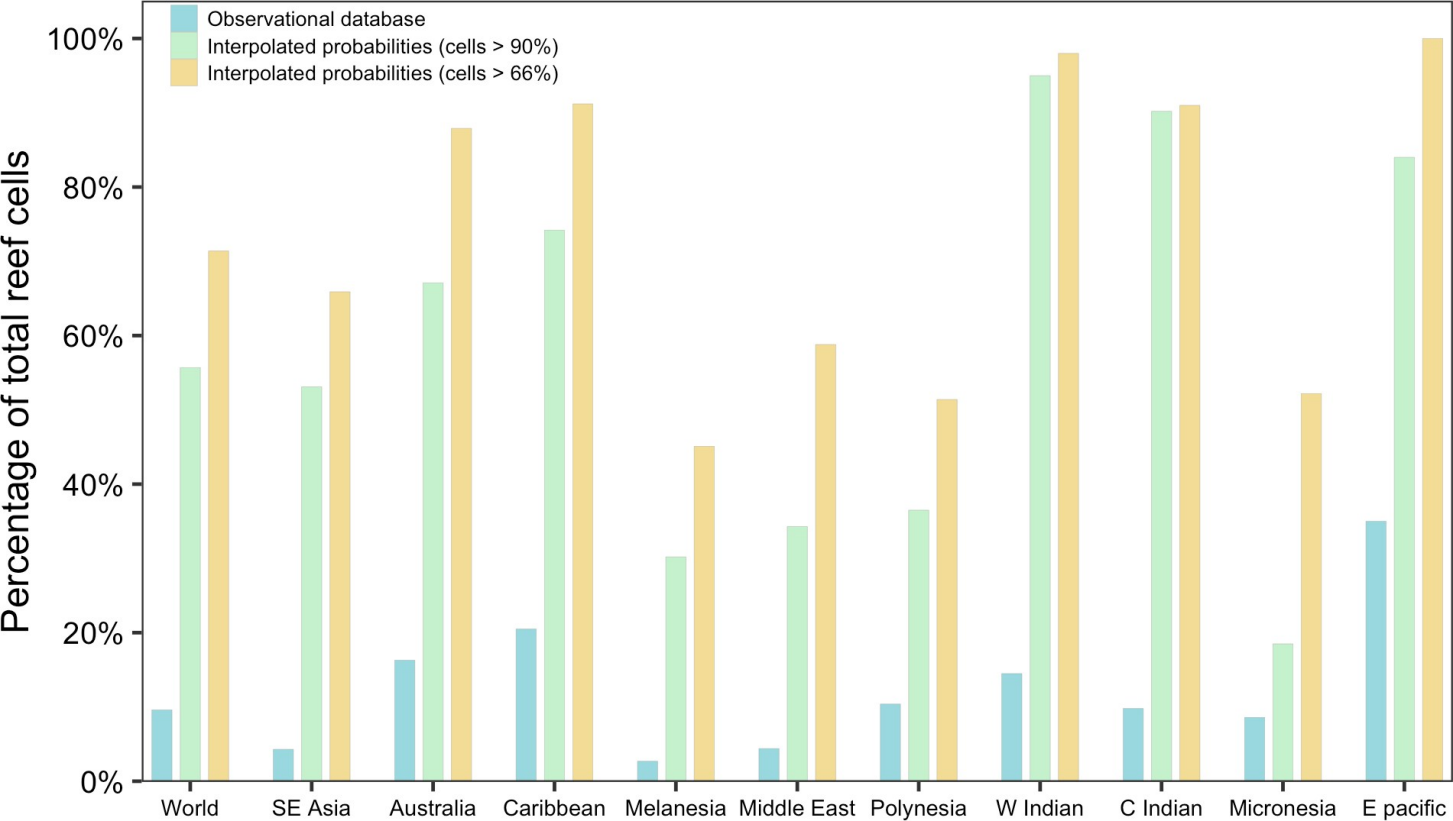
Percentage of coral cells by region with bleaching reports, >90% bleaching probability and >66% bleaching probability at least once during entire 1985–2017 period.

**Fig 3:**
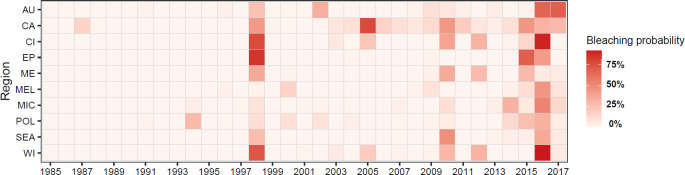
Mean bleaching probability across all coral reefs cells by year and region.

The interpolated bleaching probabilities results sharply increased the number of grid cells which likely (>66% chance) or very likely (>90% chance) experienced bleaching at least once across the 1985–2017 period compared to that shown only from the observational data (Fig 2). At the regional level, SE Asia, Australia and the Caribbean featured the greatest bleaching extent over time in terms of total number of cells; however, as a proportion of total reef cells in each region, the bleaching extent was greater in the East Pacific, West and Central Indian, and the Caribbean (74–95% of reefs very likely experienced bleaching and 91–100% likely experienced bleaching). Most regions also saw more extensive bleaching during 2014–2016 compared to the other two global event, with the exception of SE Asia, which had greater bleaching extent in the 2009–2010 event, and the East Pacific and the Middle East, which had greater bleaching extent in the 1997–1998 (S5 Fig). The mean values of the interpolated bleaching probabilities across all coral reef cells highlight the greater likelihood and extent of bleaching during the three global mass coral bleaching episodes and during the 2010s (Fig 3). The mean bleaching probability values of *all* reef cells during the most extensive bleaching years (including 1998, 2005, 2010, and 2016) ranged between 29% and 45%.

**Fig 4:**
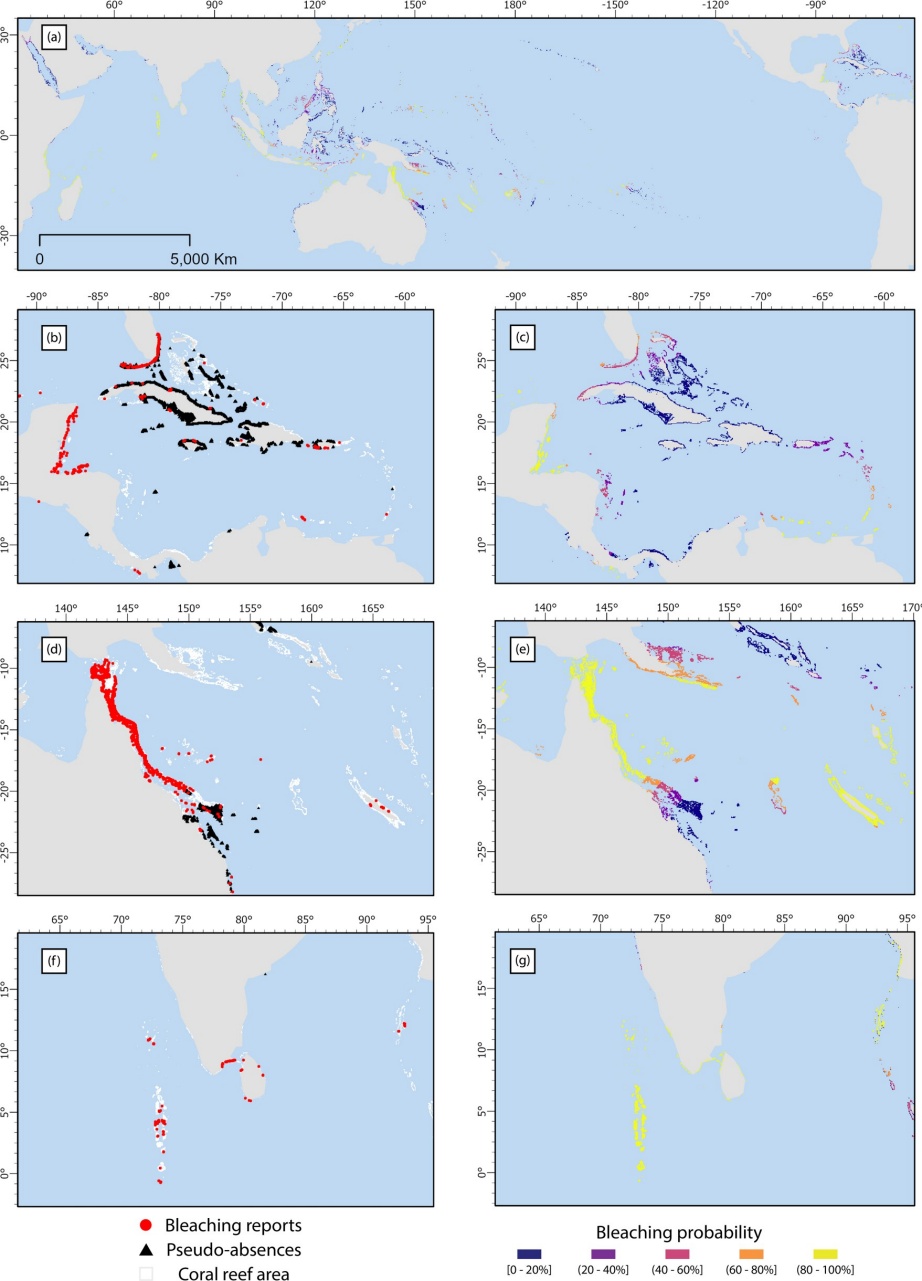
Bleaching probabilities for the year 2015, with insets for selected regions. (a) Global map; (b, d, f) Maps of observed bleaching reports and bleaching pseudo-absences used to inform kriging for the Caribbean, Great Barrier Reef and Central Indian Ocean respectively; (c, e, g) Corresponding maps of bleaching probabilities. Maps are made with the *Natural Earth* public domain vector and raster map data.

In each year of the database, the bleaching probabilities for cells containing bleaching reports were significantly higher (p<0.05) that those of all reef cells (S4 Table). The mean bleaching probability for all cells containing bleaching reports (53.9%) was significantly higher (p<0.001) than that of all reef cells (0.09%). Sample maps for the year 2015 (Fig 4) demonstrate the fit between the interpolated bleaching probabilities and the location of bleaching reports and pseudo-absences at the regional level. However, in some regions and years, like the Caribbean in 2013, although there were bleaching reports, the absence of thermal stress (DHW <0.5°C·weeks) introduced pseudo-absences in the cells with reports or adjacent to cells with reports, leading to very low bleaching probability values (see S6 Fig for sample maps).

### Historical response to thermal stress

The bleaching probabilities were positively correlated with annual maximum DHW across all years (p<0.001), and in each global event (p<0.01). The annual maximum DHW also increases significantly (p<0.01) with all the different bleaching probability subsets for all years and the global events, with the exception of the >33–50% and >50–66% groups in 2009–10 (Table 2). The mean DHW values of cells with >33% bleaching probability across all years ranged between Coral Reef Watch’s thresholds for Bleaching Alert Level 1 (4°C·weeks) and Bleaching Alert Level 2 (8°C·weeks). This range was also observed in the 2009–2010 and 2014–2016 events; however, in the 1997–1998 event, only the cells >90% bleaching probability had a mean DHW value within that range. The mean DHW values for different bleaching probability subsets varied by region (S5 Table); however, in each region, the mean DHW values of cells with very likely bleaching probability (>90%) across all years were statistically different from that of all other bleaching probability subsets.

**Table 2:** Thermal stress for reef cells with different bleaching probabilities.

Annual maximum DHW (°C·weeks)	All cells	>90%	>66–90%	>50–66%	>33–50%	>10–33%	≤10%
**All years (1985–2017)**	1.63*	6.34*	5.46*	4.44*	4.05*	3.05*	1.05*
**1997–1998**	1.39*	5.39*	3.91*	3.14*	2.94*	2.19*	0.54*
**2009–2010**	2.4*	6.22*	5.61*	4.57	5.12	3.47*	1.4*
**2014–2016**	2.96*	6.98*	6.77*	4.73*	4.23*	3.99*	1.58*

*Significantly different (p<0.01) from other bleaching probability groups for that time period, with higher probability group featuring higher mean DHW

**Fig 5:**
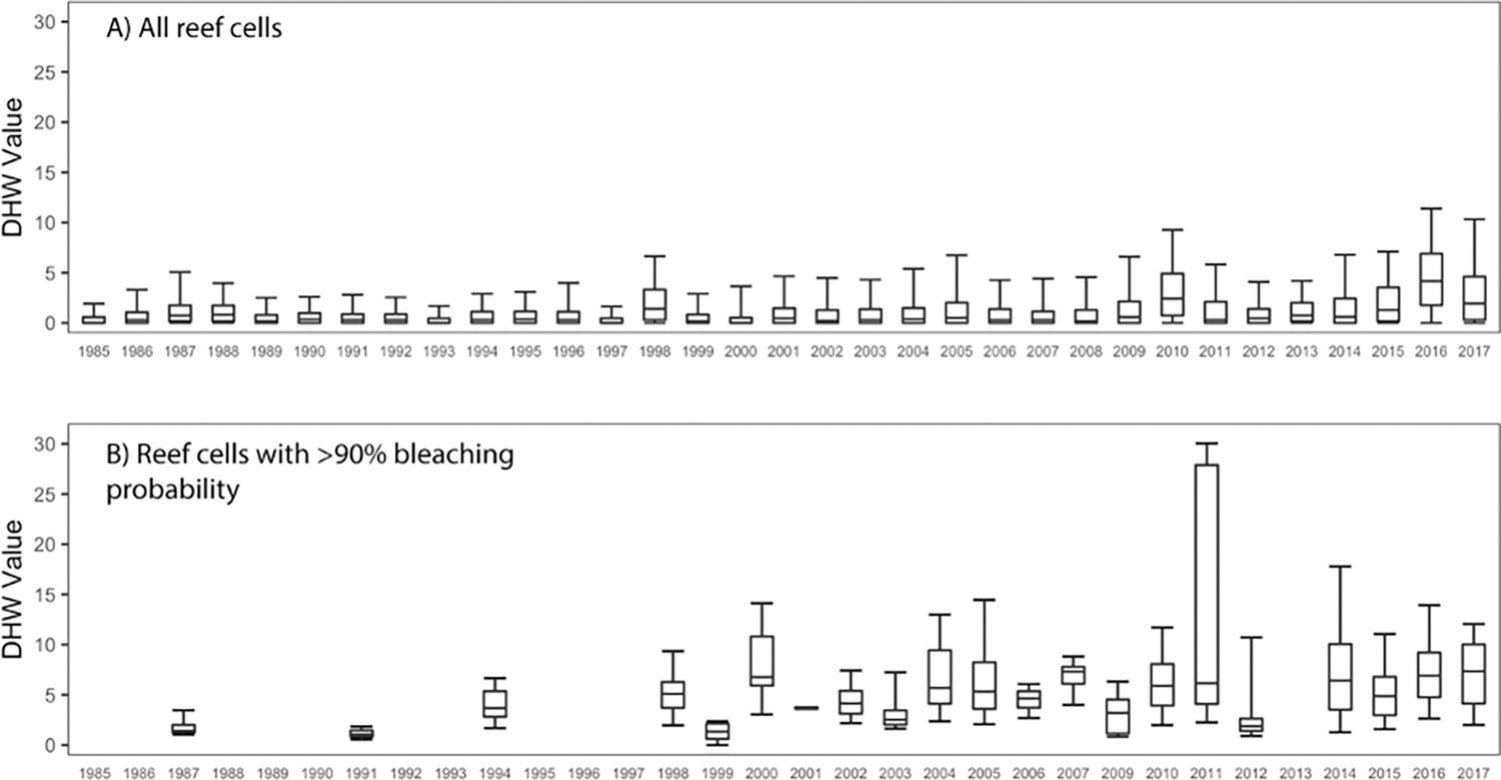
Median, quartile range, and 5th and 95th percentile of annual maximum DHW of a) all reef cells and b) reef cells with a bleaching probability above 90% 1985–2017.

To test if the bleaching threshold has increased over time, due to adaptive responses or loss of susceptible taxa, the change over the 1985–2017 period in the annual maximum DHW values of all reef cells were compared with that of cells with high bleaching probabilities. The DHW of all reef cells increased significantly (p < 0.001) by 0.06°C·weeks per year or by 1.98°C·weeks over the entire period (Fig 5A). In contrast, the DHW values of reef cells that very likely (>90% probability) experienced bleaching increased by 0.17°C·weeks (p = 0.09) per year or by 5.61°C·weeks over the entire period (Fig 5B). However, the mean DHW of reef cells that likely experienced bleaching (>66% bleaching probability) did not significantly increase.

At the regional scale, the Caribbean saw the greatest annual increase compared to the rest of the regions (2.89°C·weeks for the entire period, p < 0.001), followed by Micronesia (2.77°C·weeks, p < 0.01), Melanesia (2.3°C·weeks, p < 0.001), Australia (2.26°C·weeks, p < 0.01), Southeast Asia (1.64°C·weeks, p < 0.001), Polynesia (1.42°C·weeks, p < 0.01), and the Middle East (1.56°C·weeks, p < 0.05). Mean DHW values did not significantly increase in the Central Indian, East Pacific, or Western Indian Ocean regions. There were no significant increases in the cells that likely or very likely experienced bleaching at the regional level in the 1985–2017 period.

## Discussion

The updated observational database and interpolated bleaching probabilities capture the increasing spatial extent of bleaching over the past few decades, as well as the rare nature of mass coral bleaching before the 1990s (i.e. 91% of reports have occurred since 1990). Version 2 of the coral bleaching database includes more than three times as many positive bleaching reports as version 1. This difference reflects the recent increase in both surveying effort and in the extent of bleaching. Version 2 features 21% more positive bleaching reports than a valuable recent compilation of existing bleaching and coral reef monitoring databases [17], which points to the merit of curating global-scale databases of environmental phenomena like bleaching by regularly surveying the literature and the research community.

The new data, the infilling of past data gaps, and the use of a nearest neighbour approach for reports on land allowed for estimations of bleaching probabilities in more regions and years than in version 1. The estimated bleaching probabilities are also, on average, higher in version 2 due to the improved methodology, including the increase in data coverage, the automated model fitting technique and the optimization of the pseudo-absence DHW threshold. The results revealed that an estimate of 56–71% (a probability of likely to very likely) of the world’s reefs experienced bleaching at least once over the 1985–2017 period. This is consistent with the assessed thermal stress for global reef locations over the 1985–2017 period by [16] (>50% of reef cells). The assessment of the three global bleaching events showed that while the 1997–1998 and 2009–2010 events saw a similar bleaching extent (15.5–16.1% of reefs very likely experienced bleaching), the 2014–2016 event was between 1.8 and 2.6 times more extensive (28.5% very likely experienced bleaching and 41.6% likely experienced bleaching). These results differ from the findings in the global study by [4] that shows a comparable extent for the 1997–1998 and 2015–2016 periods (74% and 75%, respectively). However, those estimates are based on whether bleaching was observed at any sites within 100 selected reef regions (e.g., island chains) of varying sizes, whereas this analysis is based a spatially explicit analysis of smaller and more equal area 0.05° x 0.05° grid cells; the coarser resolution of the analysis by [4] leads to higher probabilities of observed bleaching.

The analysis presented here does suggest similar findings to [4] in regard to the regular bleaching occurring in the Western Atlantic (Caribbean region here) compared to other regions in the world, although recurrence was not explicitly assessed in this study. The results for the 1997–1998 event showed an estimate of 15.5% of reefs that very likely bleached and 15.6% likely bleached (Fig 4), which is comparable with the estimate of 16% of reefs affected in [37]. The comparison between global episodes is consistent with recent analysis [5, 6] that assessed the 2014–2017 episode as the most extensive and intensive in recorded history.

The increase in the number of raw observations and interpolated bleaching probability values towards the latter part of the period studied here can be explained by the increase in severity and extent of warm-season MHWs, several of which have been attributed to anthropogenic climate change [38]. These results show a clear relationship between thermal stress and high bleaching probability, with the annual maximum DHW increasing with each probability category at the global scale and in most regions (Table 2). In this study, bleaching extent was calculated considering the likely (>66%) and very likely (>90%) probabilities only. However, the annual-maximum DHW values of cells with bleaching probability >33% were on average in excess of the Bleaching Alert Level 1 threshold of 4°C·weeks (Table 2), which suggests a lower probability might be more representative of the onset of some bleaching. It should also be noted that the annual maximum DHW might exceed the thermal stress at the time of bleaching occurrence [8], such that the DHW associated with different probabilities of bleaching onset is likely lower than implied by the annual maximum value used here.

Analysis of the interpolated bleaching probabilities adds to the evidence that reef-scale bleaching thresholds may have increased on average over time [8, 9, 39], through mechanisms like changes in coral community composition and loss of susceptible taxa, coral physiological acclimation, evolution of the coral host or symbiotic algae, or shuffling towards more heat tolerant symbionts. The annual maximum DHW of reef cells that very likely (>90% probability) experienced bleaching increased by 0.17°C-week per year over the 1985–2017 period, three times the rate of all reef cells. An increase in annual maximum DHW is expected due to ocean warming, and thus is not in itself evidence of adjustment in the bleaching thresholds. However, the higher rate of change among very likely bleached reef cells in the interpolated dataset suggests that, on average, higher thermal stress may have become necessary to cause severe reef-scale bleaching over time. Additional modelling to estimate the likely dates of bleaching onset in each grid cell in the interpolated dataset would be necessary to more precisely analyse the change in bleaching thresholds and other predictive metrics over time.

This analysis of the drivers of bleaching presented here, which employs only the conventional DHW metric, is intentionally preliminary. By making the raw observational database and the interpolated bleaching probabilities publicly available, we hope the coral reef community will employ both products to explore bleaching drivers and prediction in more detail. The regional variability in the annual maximum DHW for different bleaching probabilities points to the value of using these databases and others [4, 17] to further assess regional relationships [40], real-time prediction methods [41, 42] and the role of environmental drivers which may limit bleaching response, like turbidity [43], internal waves [44], and high-frequency temperature variability [45]. The raw observational database has already been employed to provide support for the hypothesis that cloudiness can reduce susceptibility to thermal stress [46].

The observational database and the interpolated bleaching probabilities do have several key limitations. One inherent limitation of a global, retrospective analysis is that the observational data was derived using a wide range of monitoring methods, which introduces potential biases [47]. The interpolated bleaching probability data provide a more robust spatial representation of the likelihood of bleaching each year than is possible from observational data alone, which, like any database of its kind, is limited by the geographical biases in reef monitoring. Even with the improved observational coverage in this version of the database, the robustness of the interpolation results in a region is still limited by the quality of reporting and the level of surveying effort in that region. A lack of observations from a region or limited geographic spread in observations creates a challenge for the spatial interpolation; for example, the reports in 1996 in the Indian Ocean were too geographically concentrated to conduct kriging in that region that year.

The spatial interpolation is also potentially useful in verifying the accuracy of observational data. The differences in bleaching probability values between cells with bleaching observations can point to potential errors in the observations. For example, in the Caribbean in 2013, moderate to severe bleaching was reported in parts of Florida despite the annual maximum DHW not exceeding 0.5°C·weeks. Given the exposure to mid-latitude winter cold fronts in Florida, it is possible that these specific reports are due to mistimed reporting of a cold-water bleaching event [48] or paling due to disease or other factors. The discrepancy suggests that spatial interpolation products like the interpolated bleaching probabilities could be used to detect potential errors or inconsistencies in voluntary and citizen science data.

## Conclusion

This study provides an updated version of the comprehensive databases describing historical mass coral bleaching from 1963 to 2017. In addition to providing global estimates of bleaching throughout the past few decades, a regional analysis provides new insights regarding the extent of bleaching for all warm-water reef areas. The new observational data from this updated version allow for more accurate spatial modeling, providing more global maps of bleaching probability for recent and past years compared to the first version. The assessment of the updated databases shows that bleaching extent has increased through time, with a drastic increase in the 2014–2017 period. The evidence that bleaching probability increases with higher annual maximum thermal stress points to the potential applications of the database for large-scale bleaching analysis. The analysis presented here focused on the three globally distributed coral bleaching events (1997–1998, 2009–2010, and 2014–2016). However, the data provide an opportunity to analyze regional trends or regional events in more detail, like 2002 and 2011 events in Australia or the 2005 event in the Caribbean.

## Supporting information

S1 Fig Defined boundaries for the ocean region category. Black dots represent 0.05° x 0.05° cells containing coral reefs.
(DOCX)

S2 Fig Defined boundaries for the world’s reef regions. Map is adapted from Kleypas et al. (2008), with AU, Australia; CA, Caribbean/Atlantic; CI, Central Indian; EP, East Pacific; ME, Middle East; MEL, Melanesia; MIC, Micronesia; POL, Polynesia; SEA, Southeast Asia; WI, West Indian. Black dots represent 0.05° x 0.05° containing coral reefs.
(DOCX)

S3 Fig Gridded bleaching observations. Number of 0.05 x 0.05 latitude-longitude grid cells with bleaching reports by year for the 1985–2017 period in version 2 of the database. Note that severity code 1 in this figure refers to severity code -1 (unknown) or 1 (mild bleaching).
(DOCX)

S4 Fig Regional bleaching observations. Percentage of 0.05° x 0.05° grid cells in each region with at least one bleaching report since 1963, with the total number of reports in each region is listed.
(DOCX)

S5 Fig Bleaching by region during global events. Percentage of coral cells by region with observed bleaching reports, >90% bleaching probability and >66% bleaching probability during the three global coral bleaching events (1997–1998, 2009–2010, 2014–2016).
(DOCX)

S6 Fig Maps of interpolated bleaching probabilities. (DOCX)

S1 Table Coral bleaching observational database legend. (DOCX)

S2 Table Comparison of different thresholds to define pseudo-absences. Summary statistics of bleaching probabilities for cells with reports are shown.
(DOCX)

S3 Table Years and regions for which indicator kriging could be conducted. (DOCX)

S4 Table Database comparison. Results of Welch two-sample t-tests between database Version 1 (V1) and Version 2 (V2, this study) for each year and region where indicator kriging was conducted in both versions.
(DOCX)

S5 Table Thermal stress for reef cells, by region. Results are shown for cells with different ranges of bleaching probability.
(DOCX)

## References

[1] Glynn PW. Coral reef bleaching: ecological perspectives. Coral reefs. 1993 Mar;12(1):1–7.

[2] Hoegh-Guldberg O. Climate change, coral bleaching and the future of the world’s coral reefs. Marine and freshwater research. 1999;50(8):839–66.

[3] Hoegh-Guldberg O, Mumby PJ, Hooten AJ, Steneck RS, Greenfield P, Gomez E, et al. Coral reefs under rapid climate change and ocean acidification. Science. 2007 Dec 14;318(5857):1737–42. doi: 10.1126/science.1152509 18079392

[4] Hughes TP, Anderson KD, Connolly SR, Heron SF, Kerry JT, Lough JM, et al. Spatial and temporal patterns of mass bleaching of corals in the Anthropocene. Science. 2018 Jan 5;359(6371):80–3. doi: 10.1126/science.aan8048 29302011

[5] Eakin CM, Sweatman H, Brainard RE. The 2014–2017 global-scale coral bleaching event: insights and impacts. Coral Reefs. 2019 Aug;38(4):539–45.

[6] Skirving WJ, Heron SF, Marsh BL, Liu G, De La Cour JL, Geiger EF, et al. The relentless march of mass coral bleaching: a global perspective of changing heat stress. Coral reefs. 2019 Aug;38(4):547–57.

[7] Eakin CM, Morgan JA, Heron SF, Smith TB, Liu G, Alvarez-Filip L, et al. Caribbean corals in crisis: record thermal stress, bleaching, and mortality in 2005. PloS One. 2010 Nov 15;5(11):e13969. doi: 10.1371/journal.pone.0013969 21125021 PMC2981599

[8] Donner SD, Rickbeil GJ, Heron SF. A new, high-resolution global mass coral bleaching database. PLoS One. 2017 Apr 26;12(4):e0175490. doi: 10.1371/journal.pone.0175490 28445534 PMC5405922

[9] Logan CA, Dunne JP, Eakin CM, Donner SD. Incorporating adaptive responses into future projections of coral bleaching. Glob Change Biol. 2014 Jan 1;20(1):125–39. doi: 10.1111/gcb.12390 24038982

[10] van Hooidonk R, Maynard JA, Liu Y, Lee SK. Downscaled projections of Caribbean coral bleaching that can inform conservation planning. Global change biology. 2015 Sep;21(9):3389–401. doi: 10.1111/gcb.12901 25833698 PMC5008158

[11] van Hooidonk R, Maynard J, Tamelander J, Gove J, Ahmadia G, Raymundo L, et al. Local-scale projections of coral reef futures and implications of the Paris Agreement. Scientific reports. 2016 Dec 21;6(1):1–8. 28000782 10.1038/srep39666 PMC5175274

[12] McManus LC, Vasconcelos VV, Levin SA, Thompson DM, Kleypas JA, Castruccio FS, et al. Extreme temperature events will drive coral decline in the Coral Triangle. Global Change Biology. 2020 Apr;26(4):2120–33. doi: 10.1111/gcb.14972 31883173

[13] Logan CA, Dunne JP, Ryan JS, Baskett ML, Donner SD. Quantifying global potential for coral evolutionary response to climate change. Nature Climate Change. 2021 Jun;11(6):537–42.

[14] Cornwall CE, Comeau S, Kornder NA, Perry CT, van Hooidonk R, DeCarlo TM, et al. Global declines in coral reef calcium carbonate production under ocean acidification and warming. Proceedings of the National Academy of Sciences. 2021 May 25;118(21):e2015265118. doi: 10.1073/pnas.2015265118 33972407 PMC8166140

[15] Liu G, Heron S, Eakin C, Muller-Karger F, Vega-Rodriguez M, Guild L, et al. Reef-Scale Thermal Stress Monitoring of Coral Ecosystems: New 5-km Global Products from NOAA Coral Reef Watch. Remote Sens. 2014 Nov 20;6(11):11579–606.

[16] Hughes TP, Kerry JT, Connolly SR, Baird AH, Eakin CM, Heron SF, et al. Ecological memory modifies the cumulative impact of recurrent climate extremes. Nature Climate Change. 2019 Jan;9(1):40–3.

[17] van Woesik R, Kratochwill C. A global coral-bleaching database, 1980–2020. Scientific Data. 2022 Jan 20;9(1):1–7. 35058458 10.1038/s41597-022-01121-y PMC8776938

[18] Mollica NR, Cohen AL, Alpert AE, Barkley HC, Brainard RE, Carilli JE, et al. Skeletal records of bleaching reveal different thermal thresholds of Pacific coral reef assemblages. Coral Reefs. 2019 Aug;38(4):743–57.

[19] UNEP-WCMC WorldFish Centre, WRI TNC. Global distribution of warm-water coral reefs, compiled from multiple sources including the Millennium Coral Reef Mapping Project. Version 4.1. 2021. Data 10.34892/t2wk-5t34

[20] Curran PJ, Atkinson PM. Geostatistics and remote sensing. Progress in Physical Geography. 1998 Mar;22(1):61–78.

[21] Barbet‐Massin M, Jiguet F, Albert CH, Thuiller W. Selecting pseudo‐absences for species distribution models: how, where and how many?. Methods in ecology and evolution. 2012 Apr;3(2):327–38.

[22] Chefaoui RM, Lobo JM. Assessing the effects of pseudo-absences on predictive distribution model performance. Ecological modelling. 2008 Feb 10;210(4):478–86.

[23] Hengl T, Sierdsema H, Radović A, Dilo A. Spatial prediction of species’ distributions from occurrence-only records: combining point pattern analysis, ENFA and regression-kriging. Ecological modelling. 2009 Dec 24;220(24):3499–511.

[24] Glacken I, Blackney P. A practitioner’s implementation of indicator kriging. In Proceedings of a one day symposium: Beyond Ordinary Kriging 1998 Oct 30.

[25] Hengl T. A Practical Guide to Geostatistical Mapping. BPR Publishers; 2011. 270 p.

[26] Hohn M. Geostatistics and petroleum geology. Springer Science & Business Media; 1998 Nov 30.

[27] Suggett DJ, Smith DJ. Interpreting the sign of coral bleaching as friend vs. foe. Glob Chang Biol. 2011 Jan;17(1):45–55.

[28] Skirving W, Marsh B, De La Cour J, Liu G, Harris A, Maturi E, et al. Coraltemp and the coral reef watch coral bleaching heat stress product suite version 3.1. Remote Sensing. 2020 Nov 25;12(23):3856.

[29] Hiemstra PH, Pebesma EJ, Twenhöfel CJ, Heuvelink GB. Real-time automatic interpolation of ambient gamma dose rates from the Dutch radioactivity monitoring network. Computers & Geosciences. 2009 Aug 1;35(8):1711–21.

[30] Pebesma EJ. Multivariable geostatistics in S: the gstat package. Computers & geosciences. 2004 Aug 1;30(7):683–91.

[31] Lucas MP, Longman RJ, Giambelluca TW, Frazier AG, Mclean J, Cleveland SB, et al. Optimizing automated kriging to improve spatial interpolation of monthly rainfall over complex terrain. Journal of Hydrometeorology. 2022 Apr;23(4):561–72.

[32] Canli E, Loigge B, Glade T. Spatially distributed rainfall information and its potential for regional landslide early warning systems. Natural hazards. 2018 Apr;91(1):103–27.

[33] Oliver MA, Webster R. Kriging: a method of interpolation for geographical information systems. International Journal of Geographical Information System. 1990 Jul 1;4(3):313–32.

[34] Kleypas JA, Danabasoglu G, Lough JM. Potential role of the ocean thermostat in determining regional differences in coral reef bleaching events. Geophys Res Lett. 2008 Feb 9;35(3).

[35] Armstrong RA. When to use the Bonferroni correction. Ophthalmic and Physiological Optics. 2014 Sep;34(5):502–8. doi: 10.1111/opo.12131 24697967

[36] Wickham H. Ggplot2: Elegrant graphics for data analysis. Springer; 2016.

[37] Wilkinson C. Status of coral reefs of the world: 2000. Australian Institute of Marine Science; 2000.

[38] Laufkötter C, Zscheischler J, Frölicher TL. High-impact marine heatwaves attributable to human-induced global warming. Science. 2020 Sep 25;369(6511):1621–5. doi: 10.1126/science.aba0690 32973027

[39] Bay RA, Rose NH, Logan CA, Palumbi SR. Genomic models predict successful coral adaptation if future ocean warming rates are reduced. Science Advances. 2017 Nov 1;3(11):e1701413. doi: 10.1126/sciadv.1701413 29109975 PMC5665595

[40] McClanahan TR, Maina JM, Darling ES, Guillaume MM, Muthiga NA, D’agata S, et al. Large geographic variability in the resistance of corals to thermal stress. Global Ecology and Biogeography. 2020 Dec;29(12):2229–47.

[41] DeCarlo TM. Treating coral bleaching as weather: a framework to validate and optimize prediction skill. PeerJ. 2020 Jul 3;8:e9449. doi: 10.7717/peerj.9449 32685288 PMC7337031

[42] Lachs L, Bythell JC, East HK, Edwards AJ, Mumby PJ, Skirving WJ, et al. Fine-tuning heat stress algorithms to optimise global predictions of mass coral bleaching. Remote Sensing. 2021 Jul 7;13(14):2677.

[43] Sully S, van Woesik R. Turbid reefs moderate coral bleaching under climate‐related temperature stress. Global Change Biology. 2020 Mar;26(3):1367–73. doi: 10.1111/gcb.14948 31912964 PMC7079097

[44] Storlazzi CD, Cheriton OM, Van Hooidonk R, Zhao Z, Brainard R. Internal tides can provide thermal refugia that will buffer some coral reefs from future global warming. Scientific reports. 2020 Aug 10;10(1):1–9. 32778666 10.1038/s41598-020-70372-9 PMC7417736

[45] Safaie A, Silbiger NJ, McClanahan TR, Pawlak G, Barshis DJ, Hench JL, et al. High frequency temperature variability reduces the risk of coral bleaching. Nature communications. 2018 Apr 26;9(1):1–2. 10.1038/s41467-018-04074-2 PMC5920114 29700296

[46] Gonzalez‐Espinosa PC, Donner SD. Cloudiness reduces the bleaching response of coral reefs exposed to heat stress. Global Change Biology. 2021 Aug;27(15):3474–86. doi: 10.1111/gcb.15676 33964101

[47] Vallès H, Oxenford HA, Henderson A. Switching between standard coral reef benthic monitoring protocols is complicated: proof of concept. PeerJ. 2019 Dec 3;7:e8167. doi: 10.7717/peerj.8167 31824774 PMC6896942

[48] Colella MA, Ruzicka RR, Kidney JA, Morrison JM, Brinkhuis VB. Cold-water event of January 2010 results in catastrophic benthic mortality on patch reefs in the Florida Keys. Coral reefs. 2012 Jun;31(2):621–32.

