# Supplementary material for: Increase in the extent of mass coral bleaching over the past half-century, based on an updated global database
Source: PLoS One. 2023 Feb 13;18(2):e0281719. doi: 10.1371/journal.pone.0281719 (PMC9925063; doi:10.1371/journal.pone.0281719)

**S1 Figure. Defined boundaries for the Ocean Region category**. Black dots represent 0.05° x 0.05° cells containing coral reef


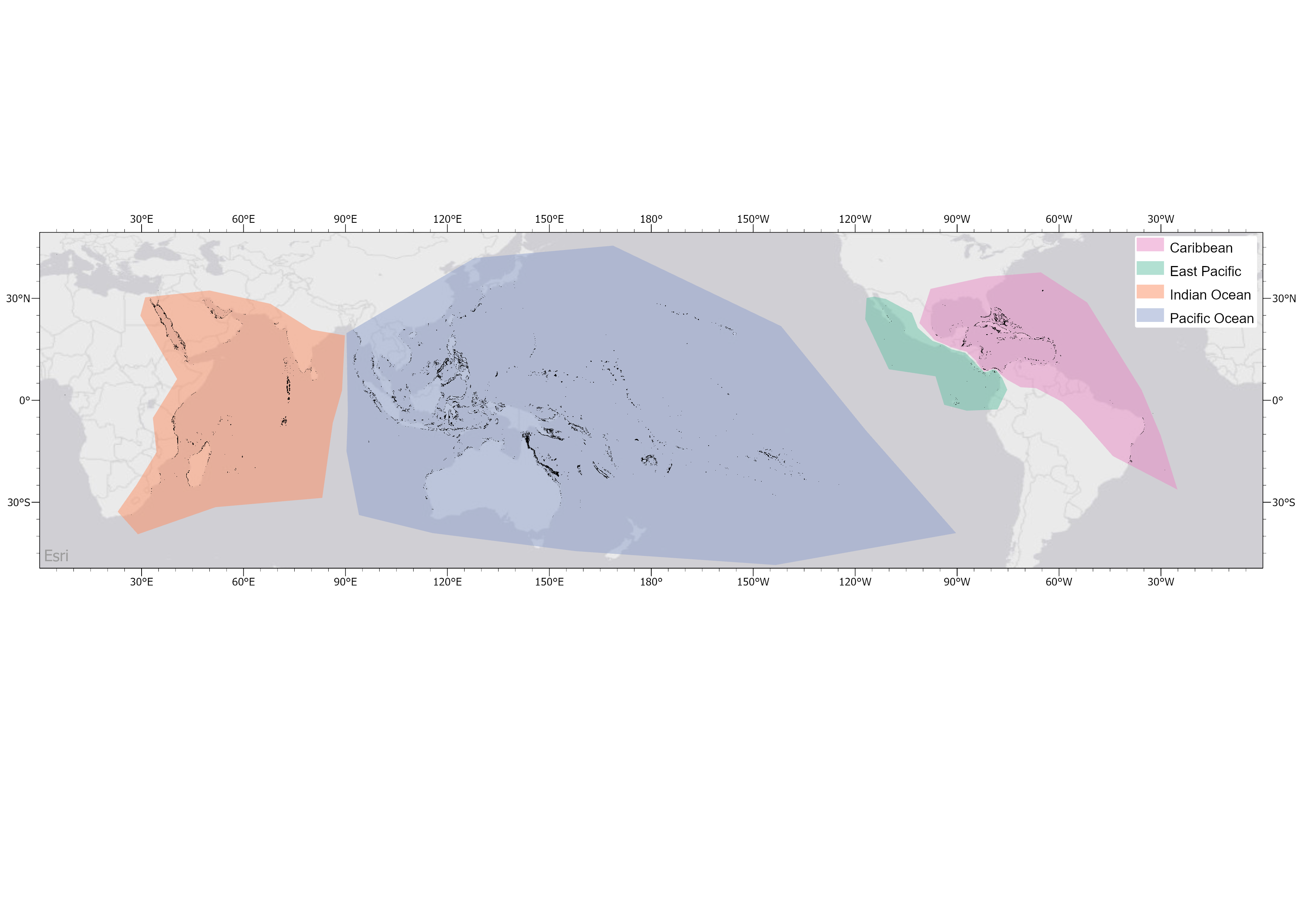

Supplement: S1 Fig — Black dots represent 0.05° x 0.05° cells containing coral reefs. (DOCX) [file pone.0281719.s001.docx]
