# Supplementary material for: Increase in the extent of mass coral bleaching over the past half-century, based on an updated global database
Source: PLoS One. 2023 Feb 13;18(2):e0281719. doi: 10.1371/journal.pone.0281719 (PMC9925063; doi:10.1371/journal.pone.0281719)

S2 Figure. Defined boundaries for the world's reef regions. Map is adapted from Kleypas et al. (2008), with AU, Australia; CA, Caribbean/Atlantic; CI, Central Indian; EP, East Pacific; ME, Middle East; MEL, Melanesia; MIC, Micronesia; POL, Polynesia; SEA, Southeast Asia; WI, West Indian. Black dots represent 0.05° x 0.05° containing coral reefs.


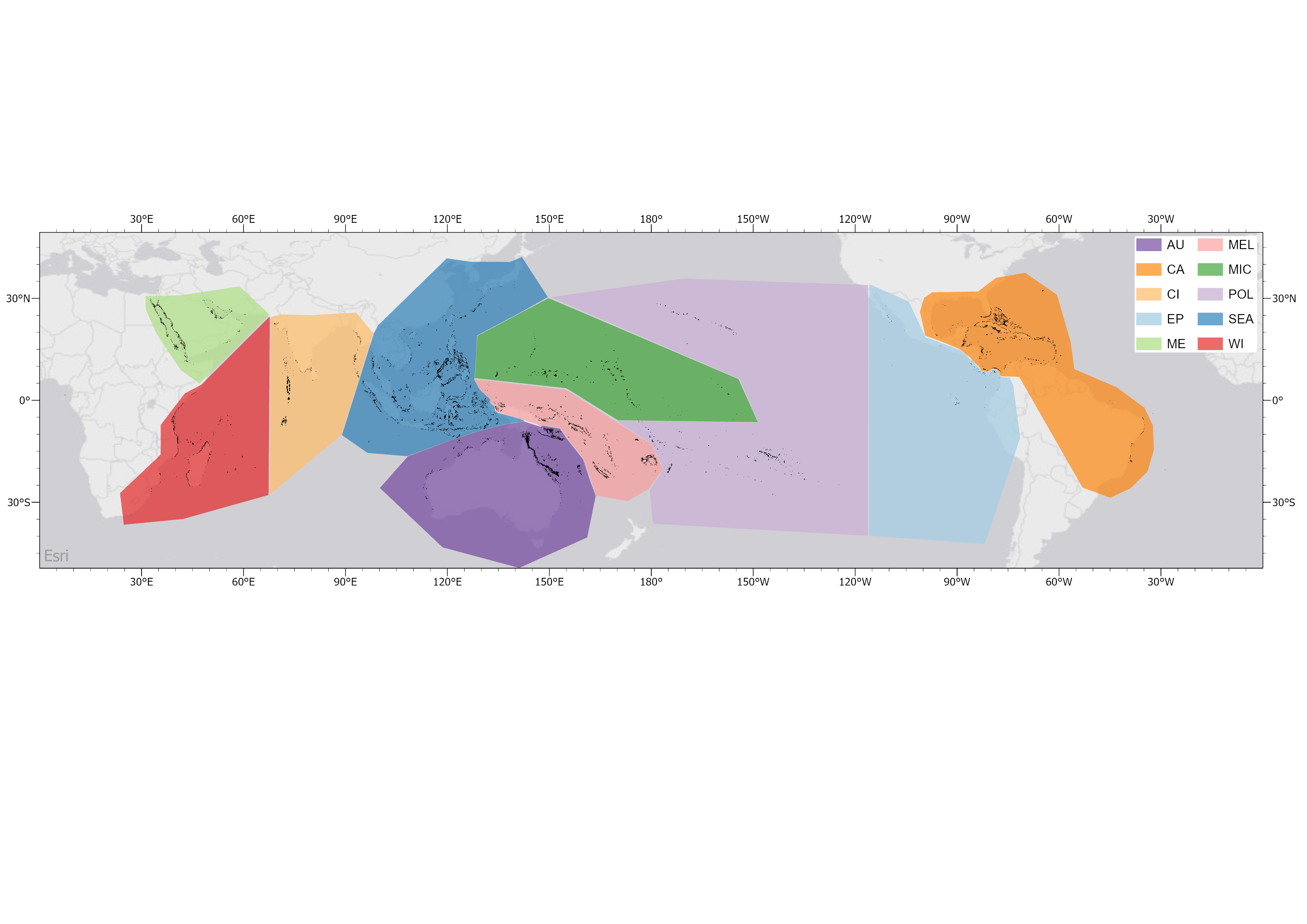

Supplement: S2 Fig — Map is adapted from Kleypas et al. (2008), with AU, Australia; CA, Caribbean/Atlantic; CI, Central Indian; EP, East Pacific; ME, Middle East; MEL, Melanesia; MIC, Micronesia; POL, Polynesia; SEA, Southeast Asia; WI, West Indian. Black dots represent 0.05° x 0.05° containing coral reefs. (DOCX) [file pone.0281719.s002.docx]
