# Supplementary material for: Increase in the extent of mass coral bleaching over the past half-century, based on an updated global database
Source: PLoS One. 2023 Feb 13;18(2):e0281719. doi: 10.1371/journal.pone.0281719 (PMC9925063; doi:10.1371/journal.pone.0281719)

**S3 Fig. Gridded bleaching observations**. Number of 0.05 x 0.05 latitude-longitude grid cells with bleaching reports by year for the 1985*–*2017 period in version 2 of the database. Note that severity code 1 in this figure refers to severity code -1 (unknown) or 1 (mild bleaching).


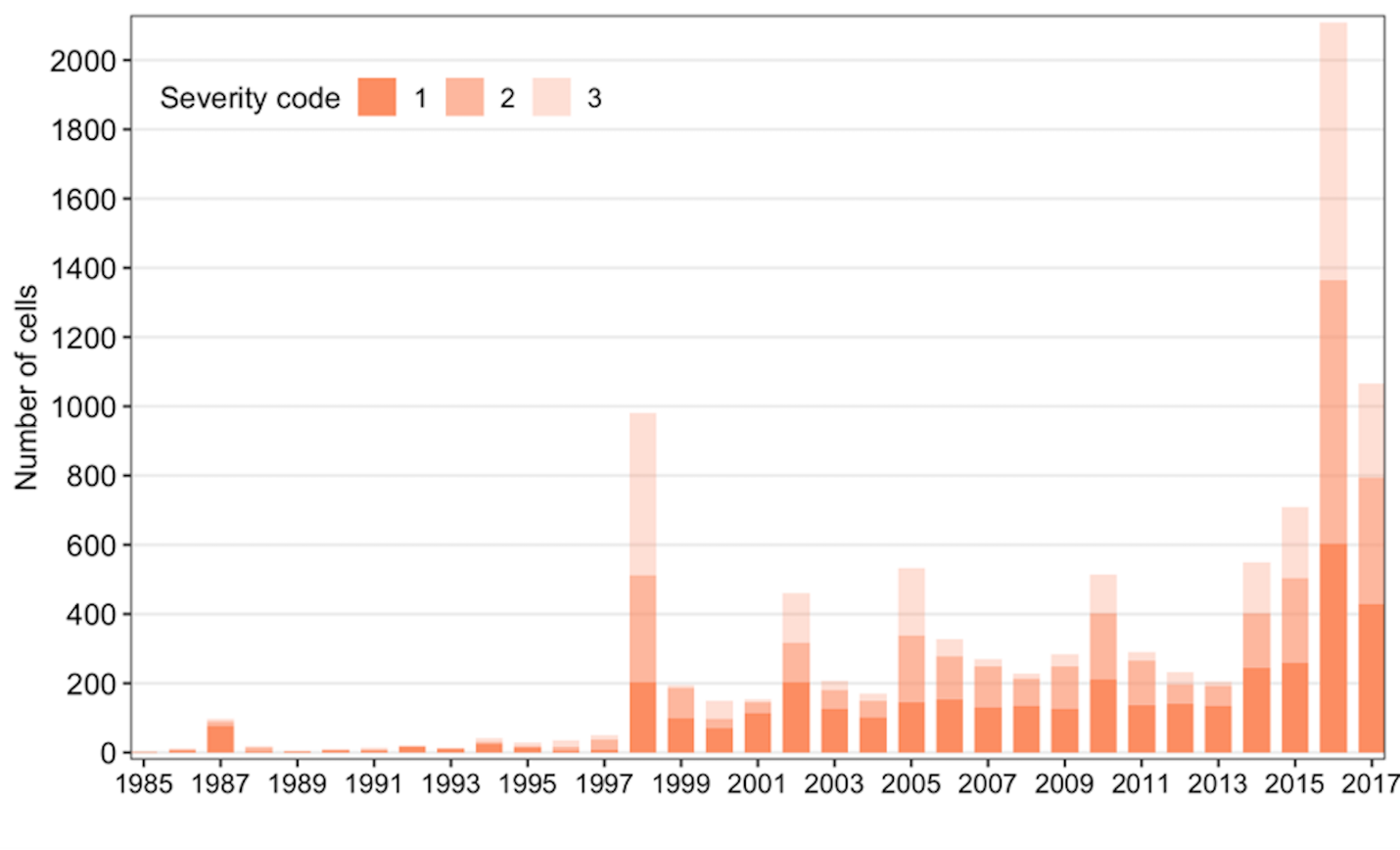

Supplement: S3 Fig — Number of 0.05 x 0.05 latitude-longitude grid cells with bleaching reports by year for the 1985–2017 period in version 2 of the database. Note that severity code 1 in this figure refers to severity code -1 (unknown) or 1 (mild bleaching). (DOCX) [file pone.0281719.s003.docx]
