# Supplementary material for: Increase in the extent of mass coral bleaching over the past half-century, based on an updated global database
Source: PLoS One. 2023 Feb 13;18(2):e0281719. doi: 10.1371/journal.pone.0281719 (PMC9925063; doi:10.1371/journal.pone.0281719)

S4 Fig. Regional bleaching observations. Percentage of 0.05° x 0.05° grid cells in each region with at least one bleaching report since 1963, with the total number of reports in each region is listed.


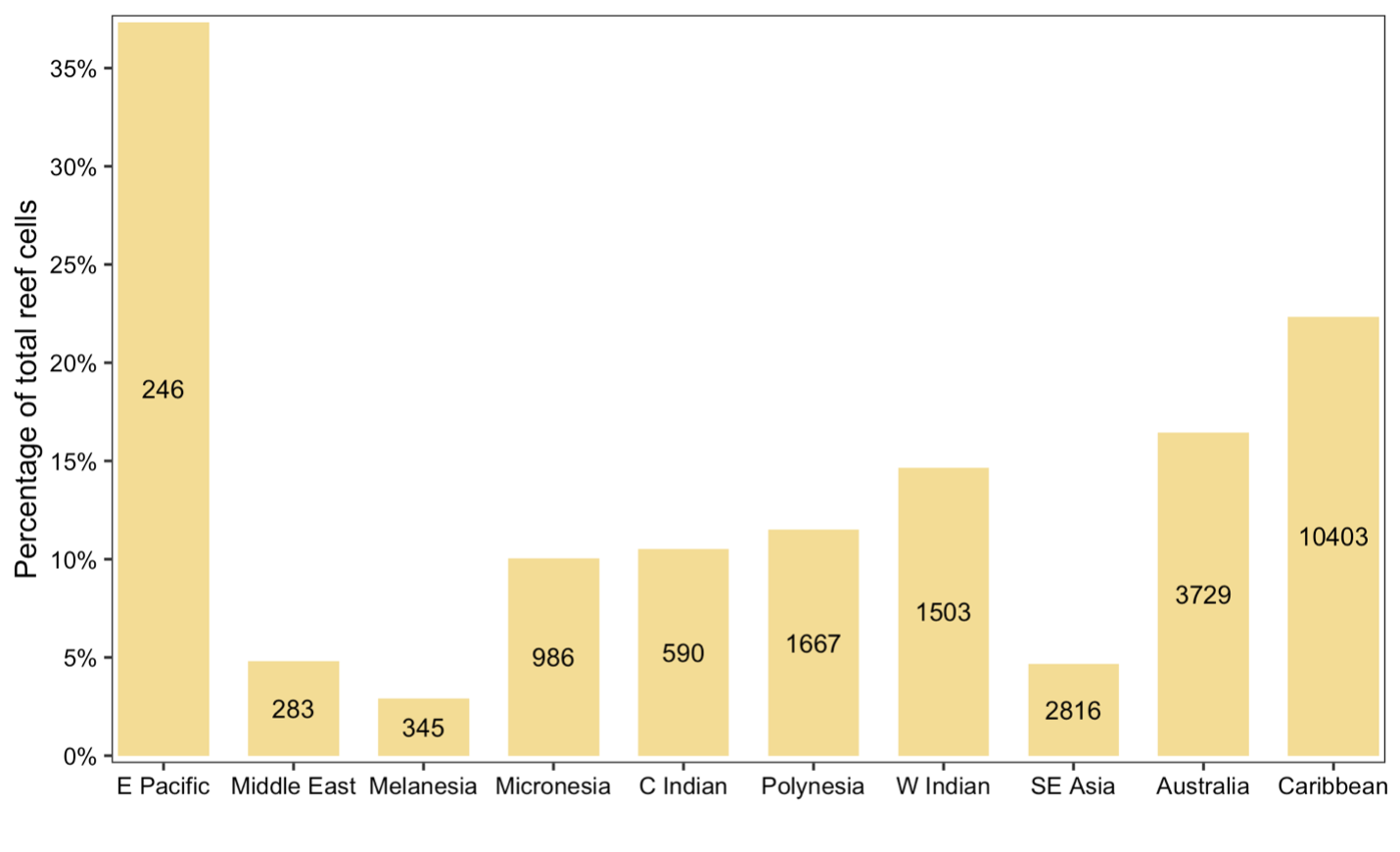

Supplement: S4 Fig — Percentage of 0.05° x 0.05° grid cells in each region with at least one bleaching report since 1963, with the total number of reports in each region is listed. (DOCX) [file pone.0281719.s004.docx]
