# Supplementary material for: Increase in the extent of mass coral bleaching over the past half-century, based on an updated global database
Source: PLoS One. 2023 Feb 13;18(2):e0281719. doi: 10.1371/journal.pone.0281719 (PMC9925063; doi:10.1371/journal.pone.0281719)

**S4 Fig. Bleaching by region during global events.** Percentage of coral cells by region with observed bleaching reports, >90% bleaching probability and >66% bleaching probability during the three global coral bleaching events (1997*–*1998, 2009*–*2010, 2014*–*2016).


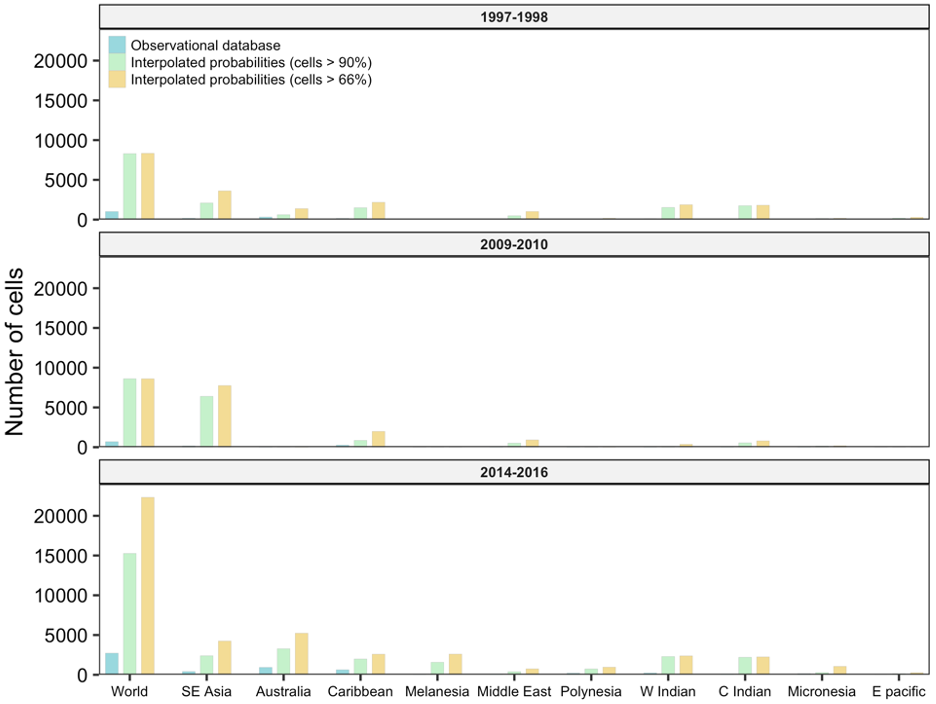

Supplement: S5 Fig — Percentage of coral cells by region with observed bleaching reports, >90% bleaching probability and >66% bleaching probability during the three global coral bleaching events (1997–1998, 2009–2010, 2014–2016). (DOCX) [file pone.0281719.s005.docx]
