# Supplementary material for: Increase in the extent of mass coral bleaching over the past half-century, based on an updated global database
Source: PLoS One. 2023 Feb 13;18(2):e0281719. doi: 10.1371/journal.pone.0281719 (PMC9925063; doi:10.1371/journal.pone.0281719)

**S6 Figure. Maps of Interpolated Bleaching Probabilities.**

A series of sample regional maps of output from the spatial interpolation are provided. Each map shows the bleaching probabilities for a region of either the central Caribbean, central Indian Ocean, or part of the western Pacific Ocean and/or Great Barrier Reef in a given year. The years and regions selected include periods with widespread bleaching, localized bleaching and minimal or no bleaching, for comparative purposes.


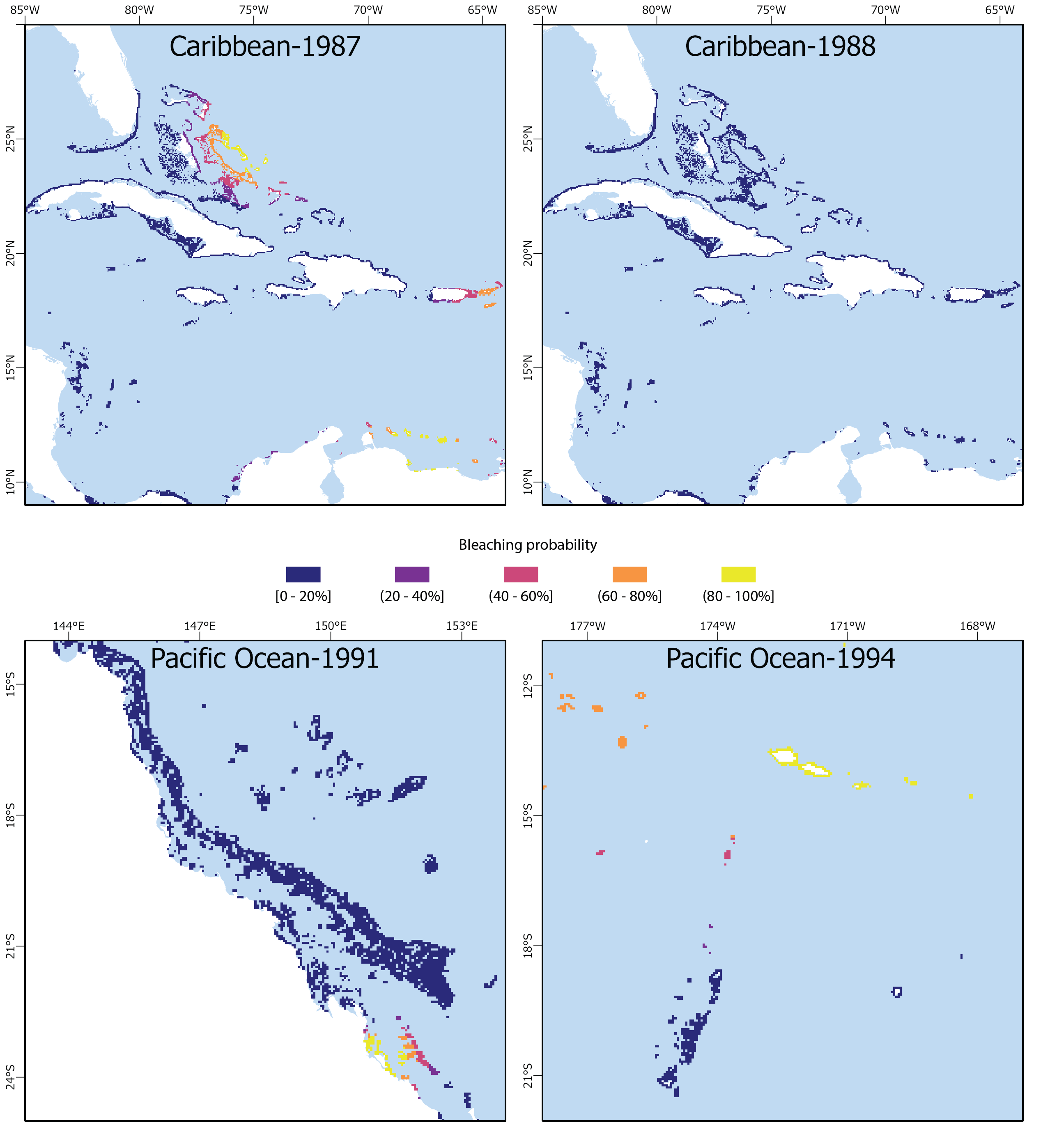


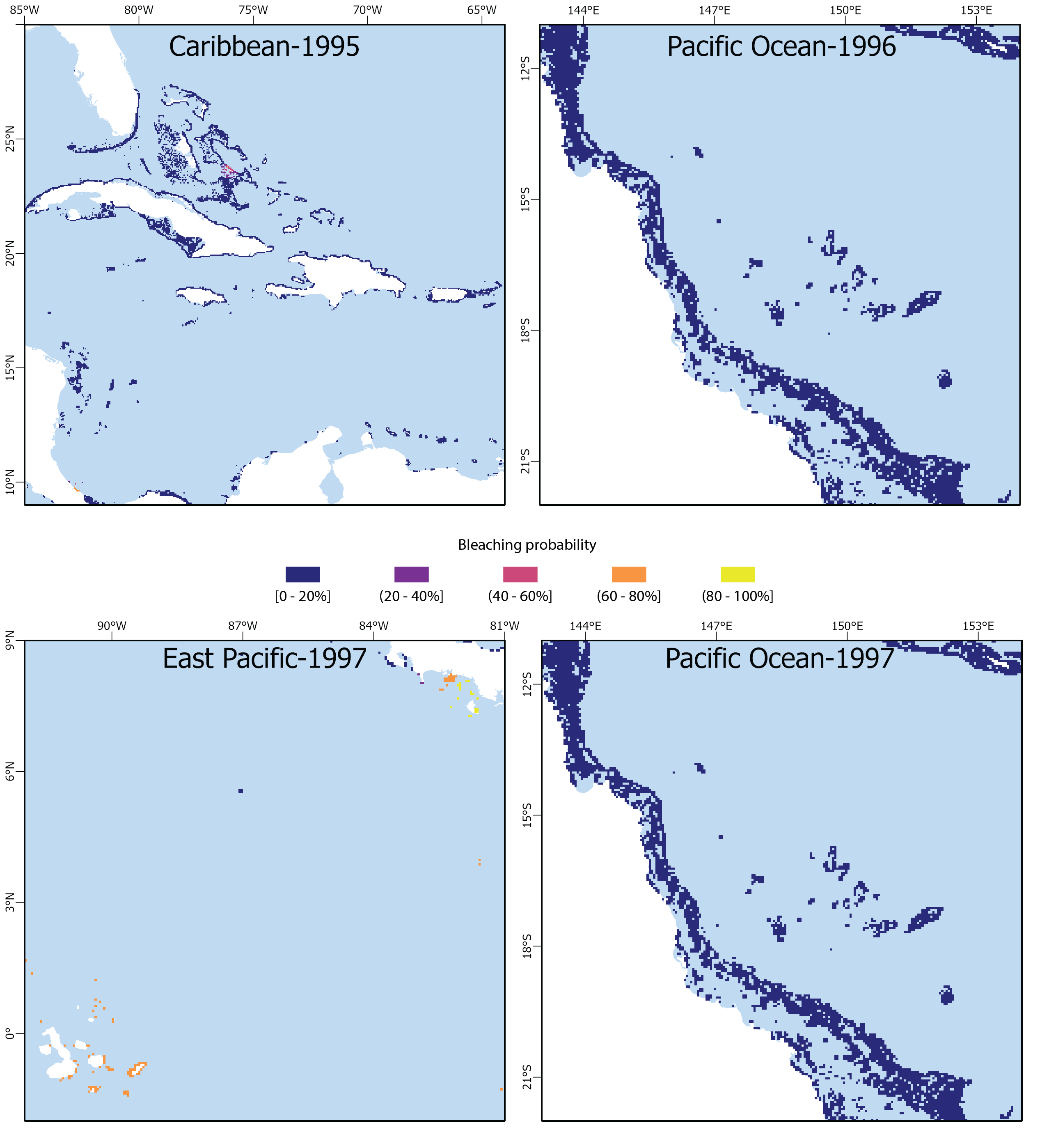


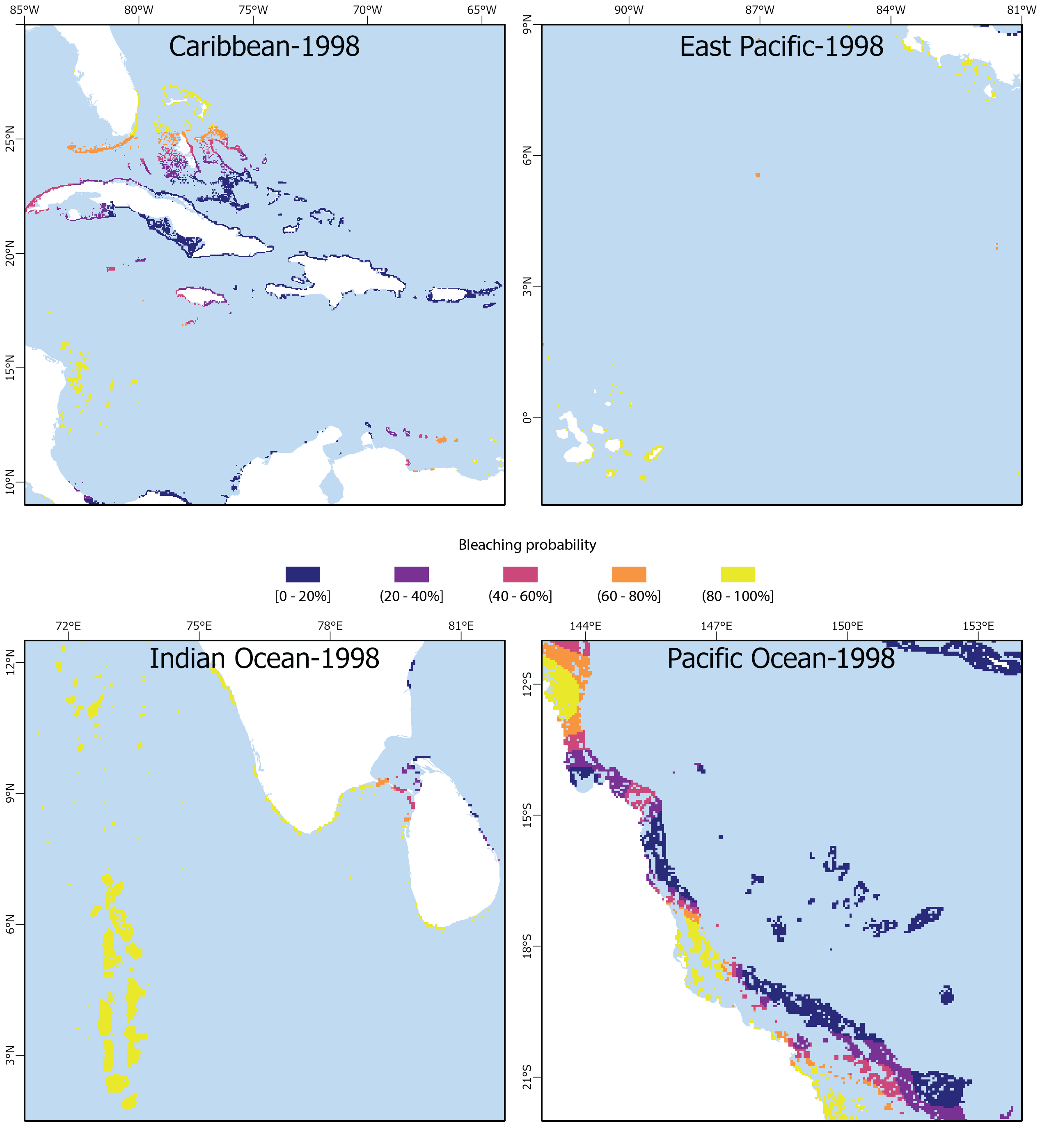


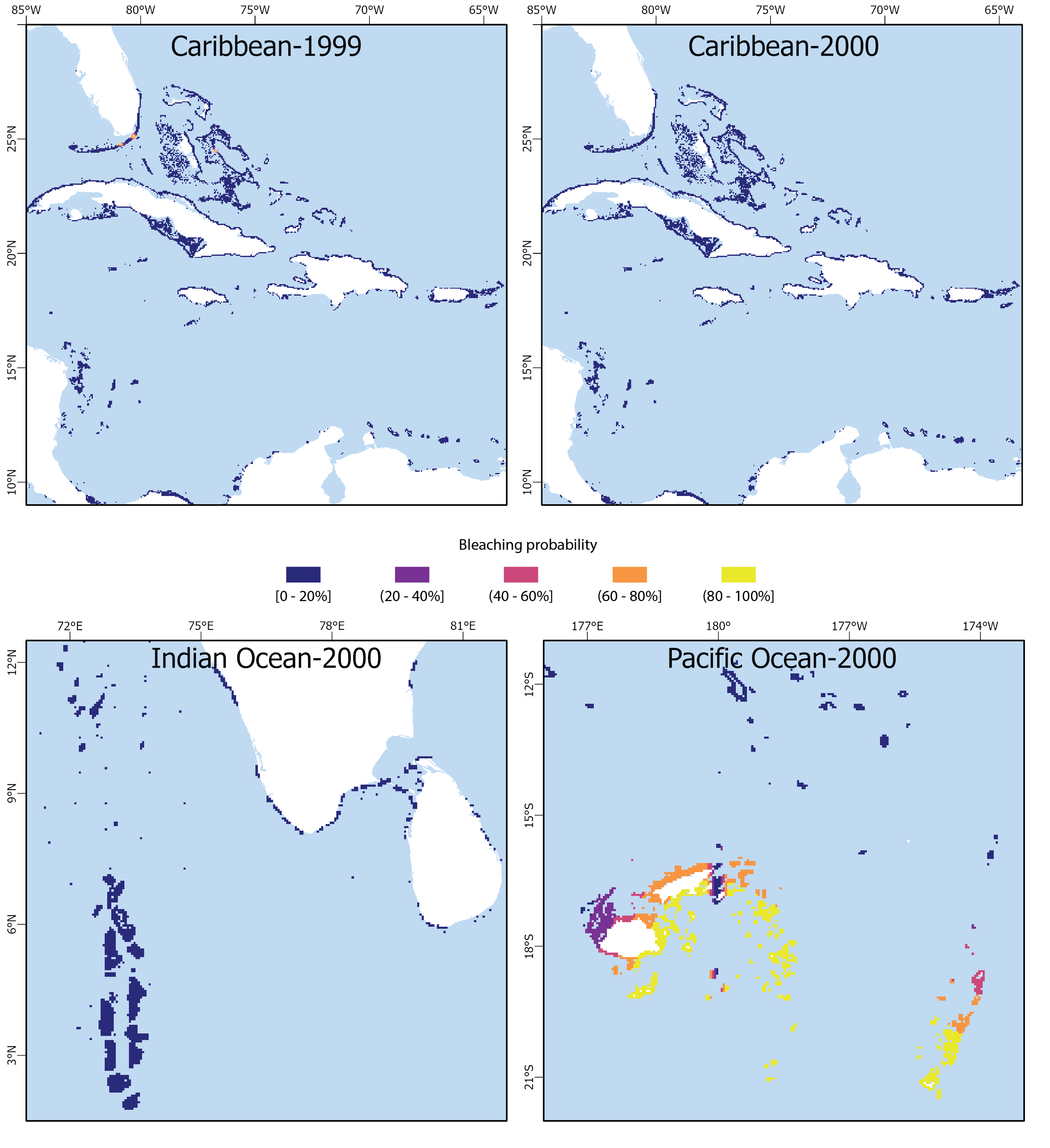


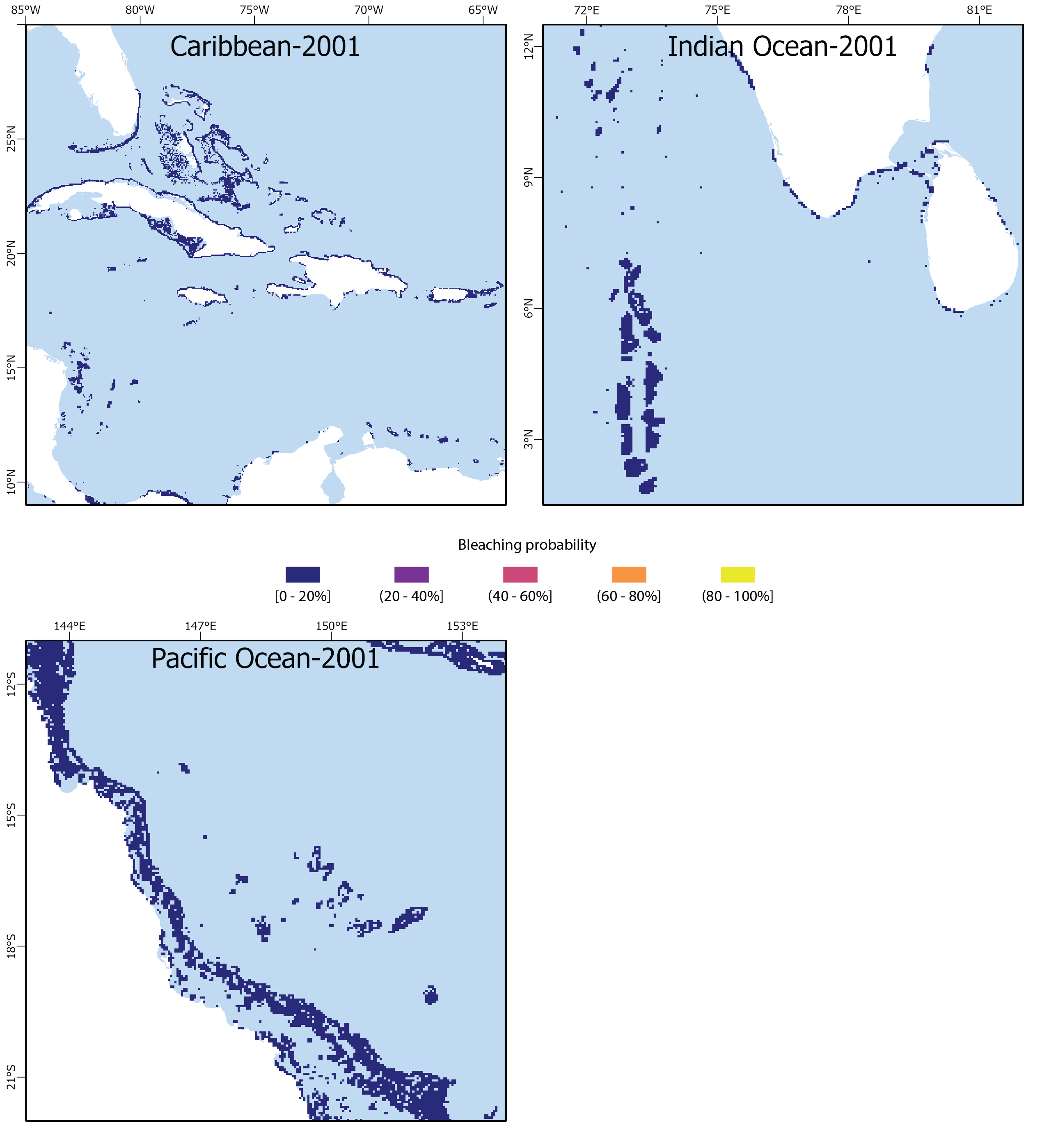


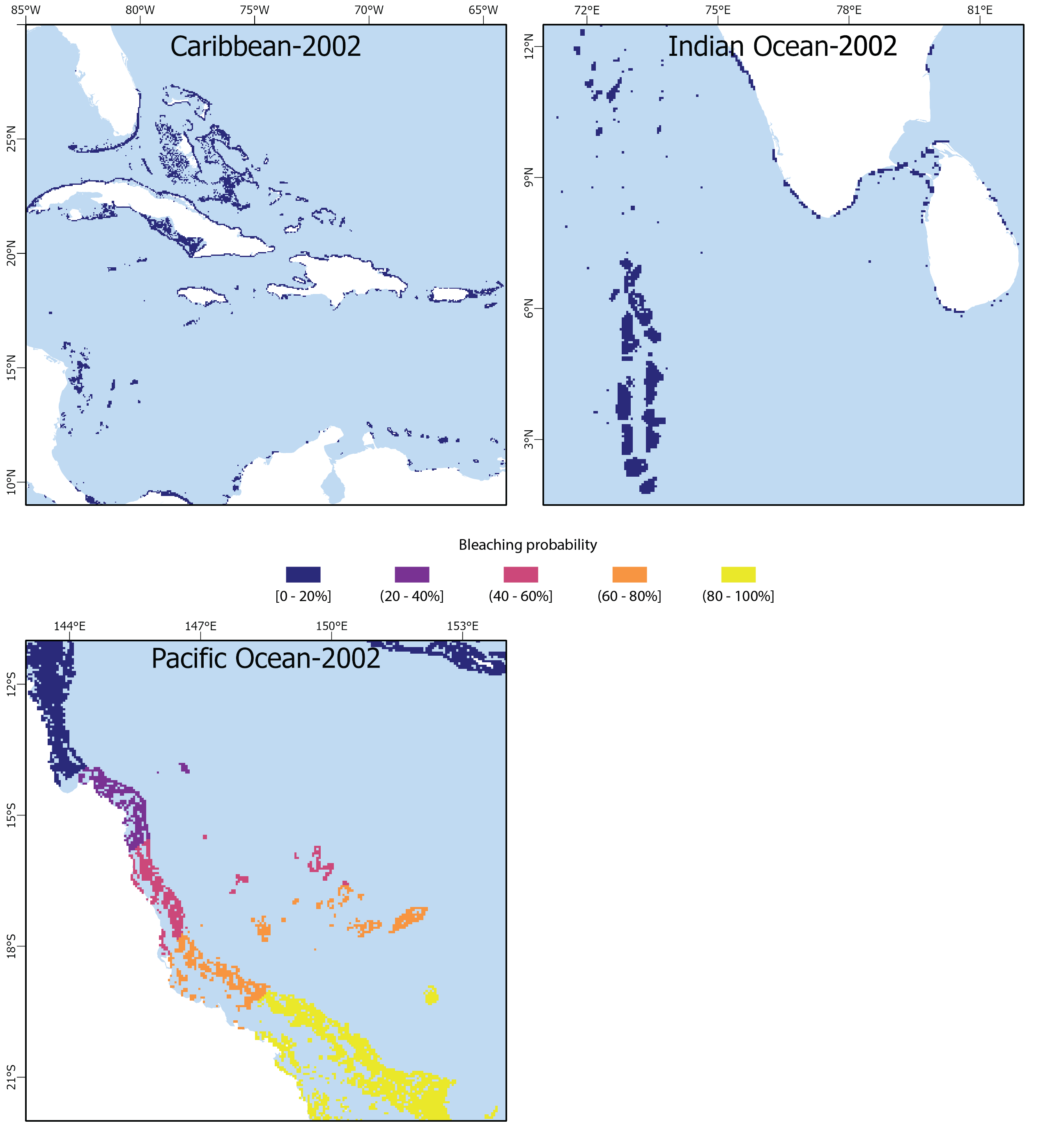


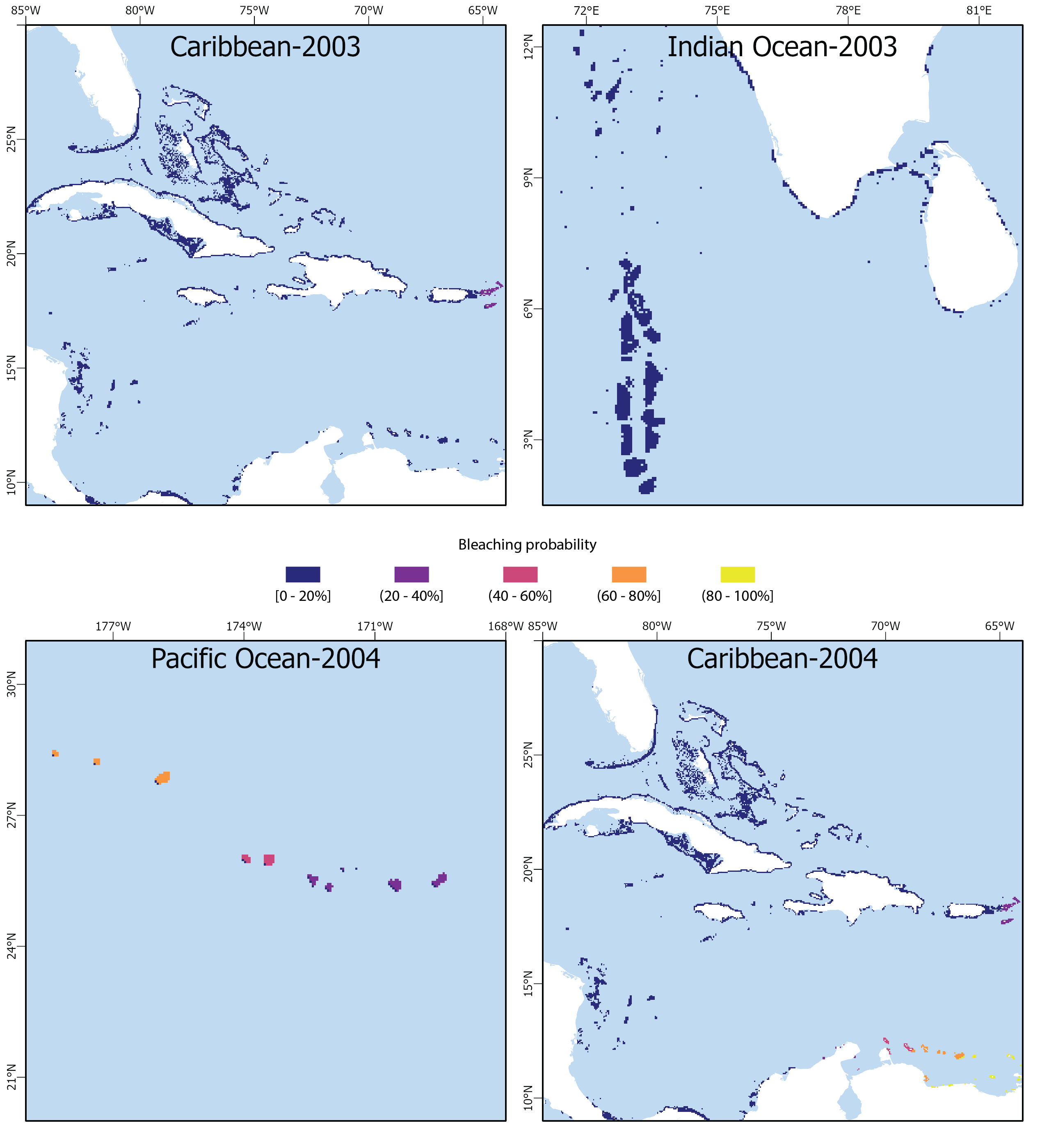


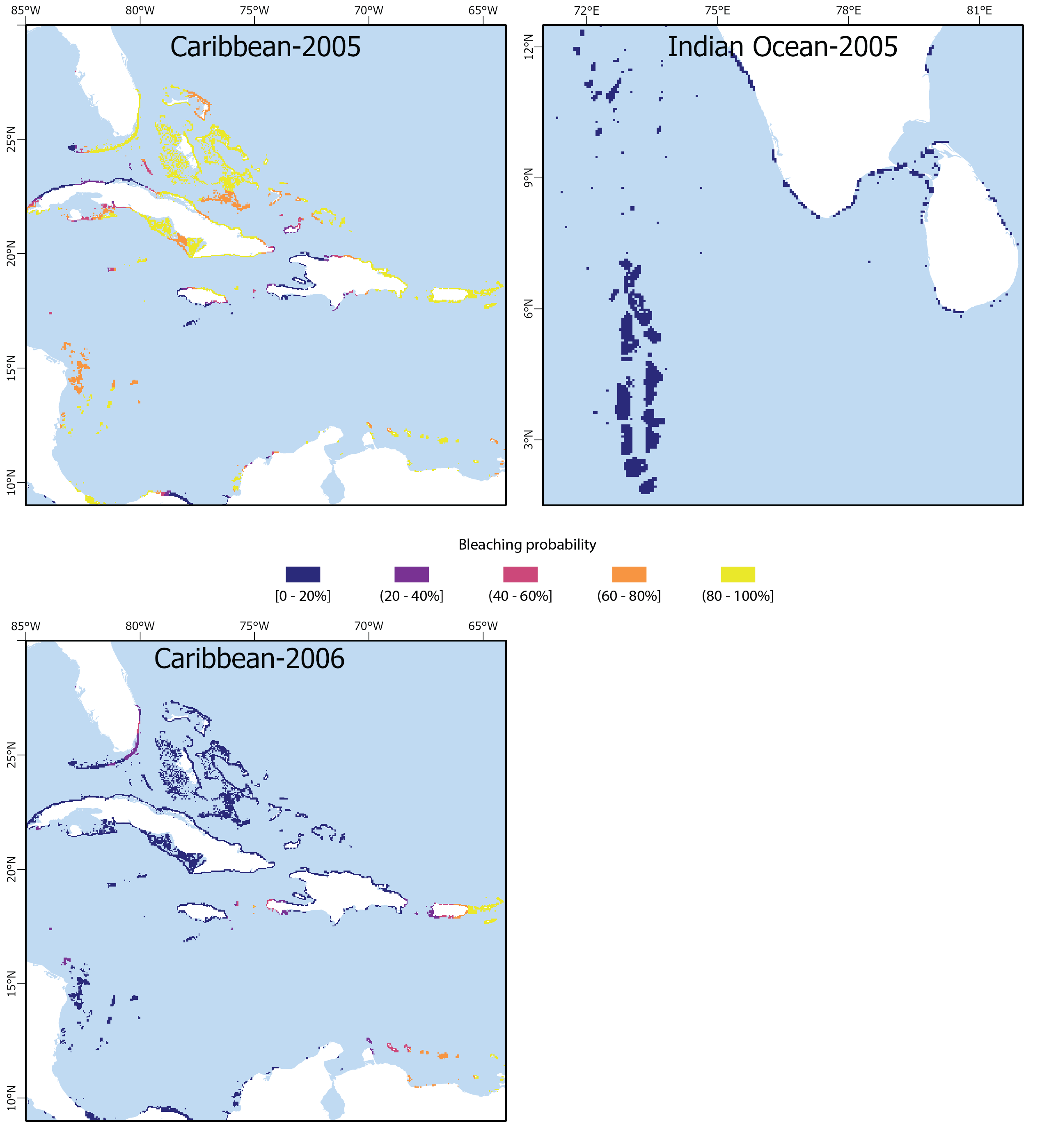


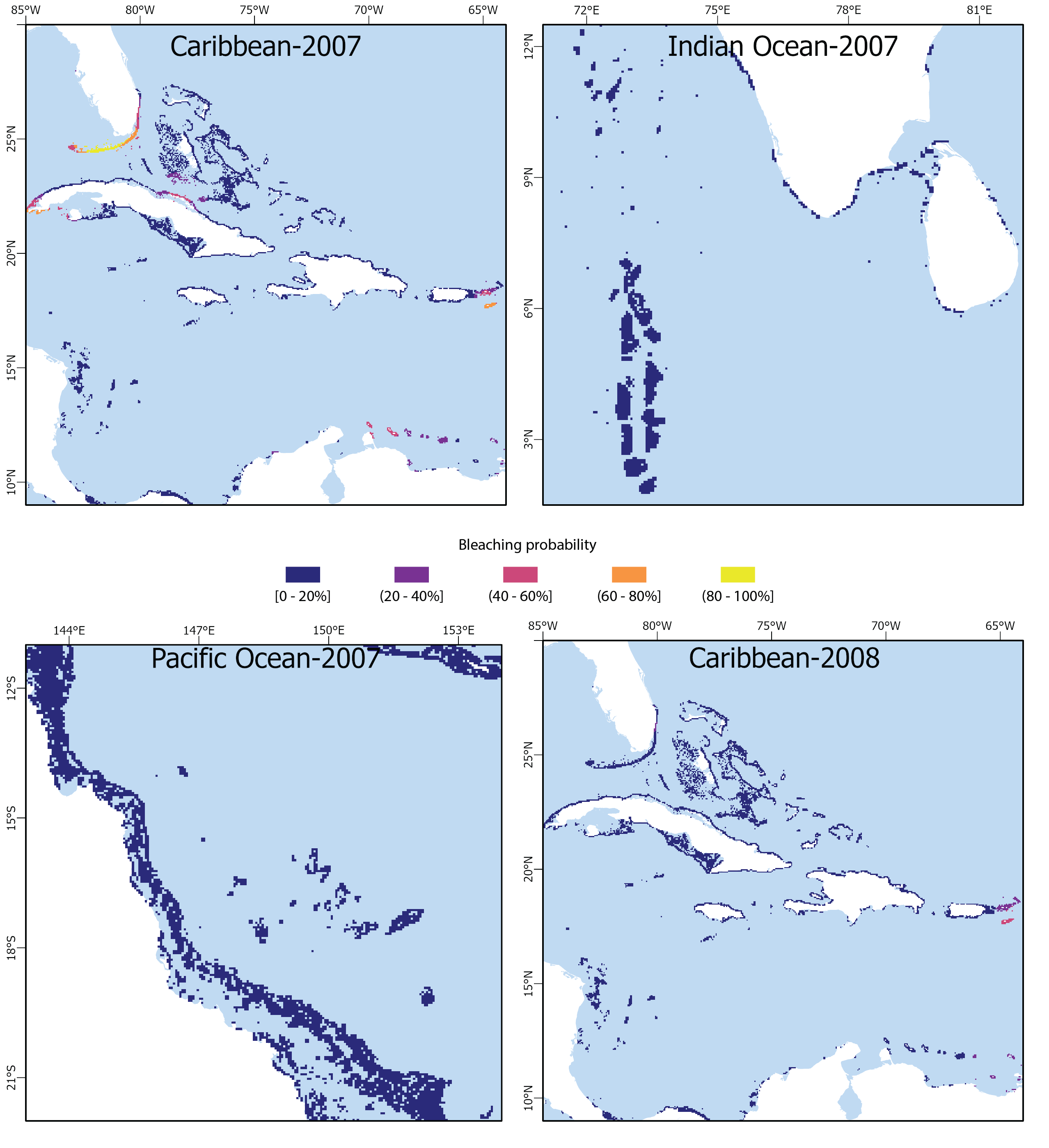


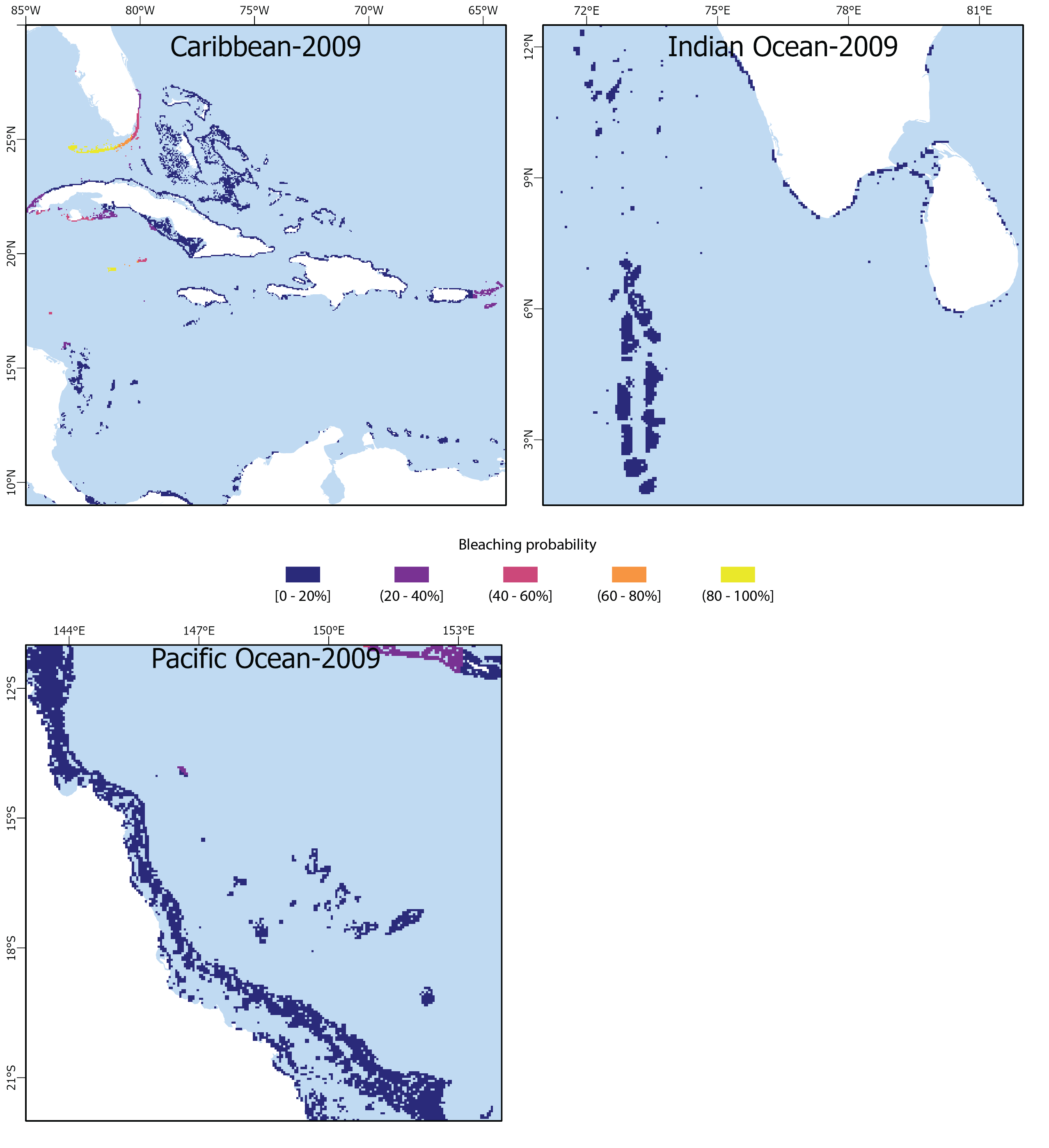


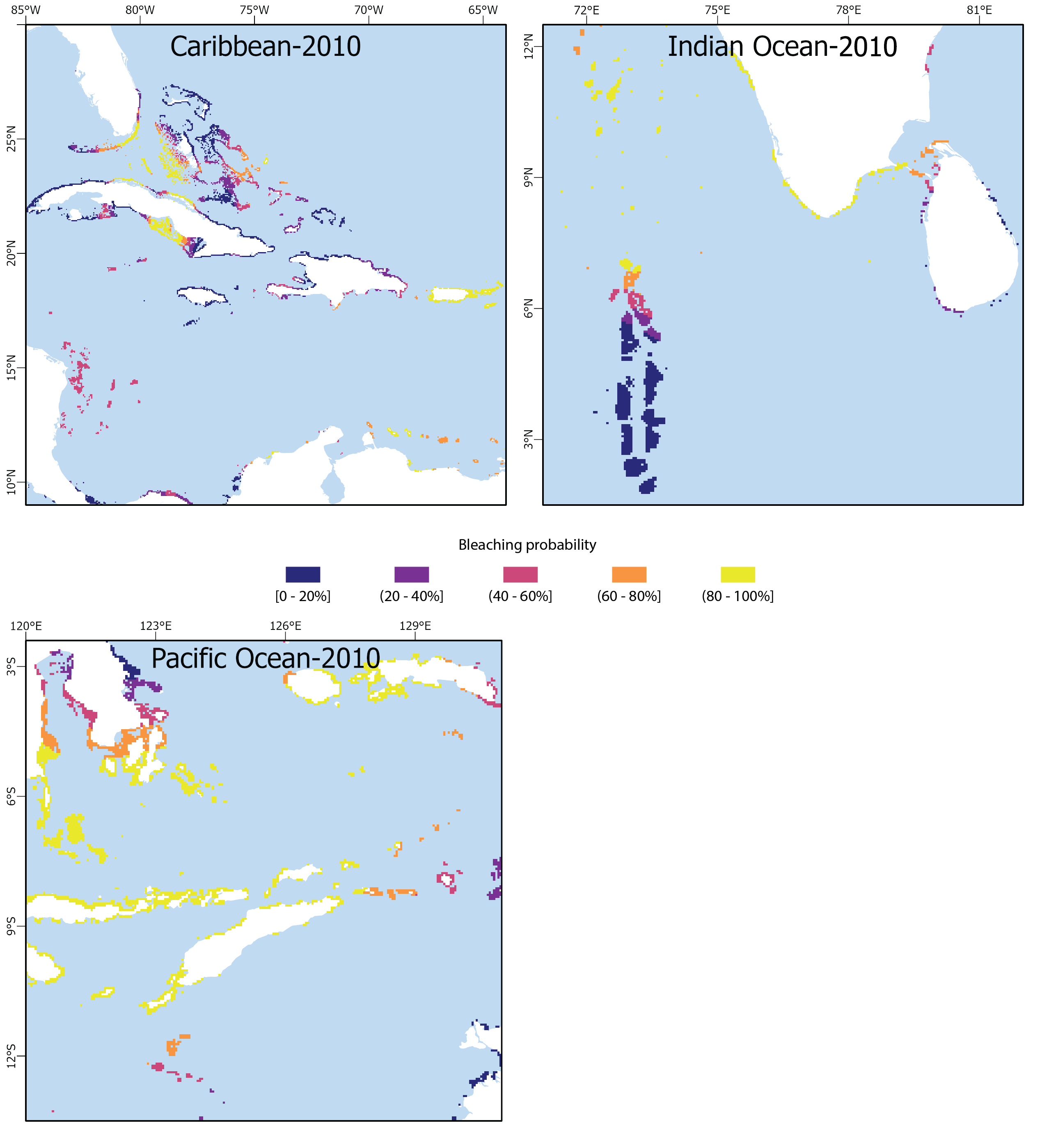


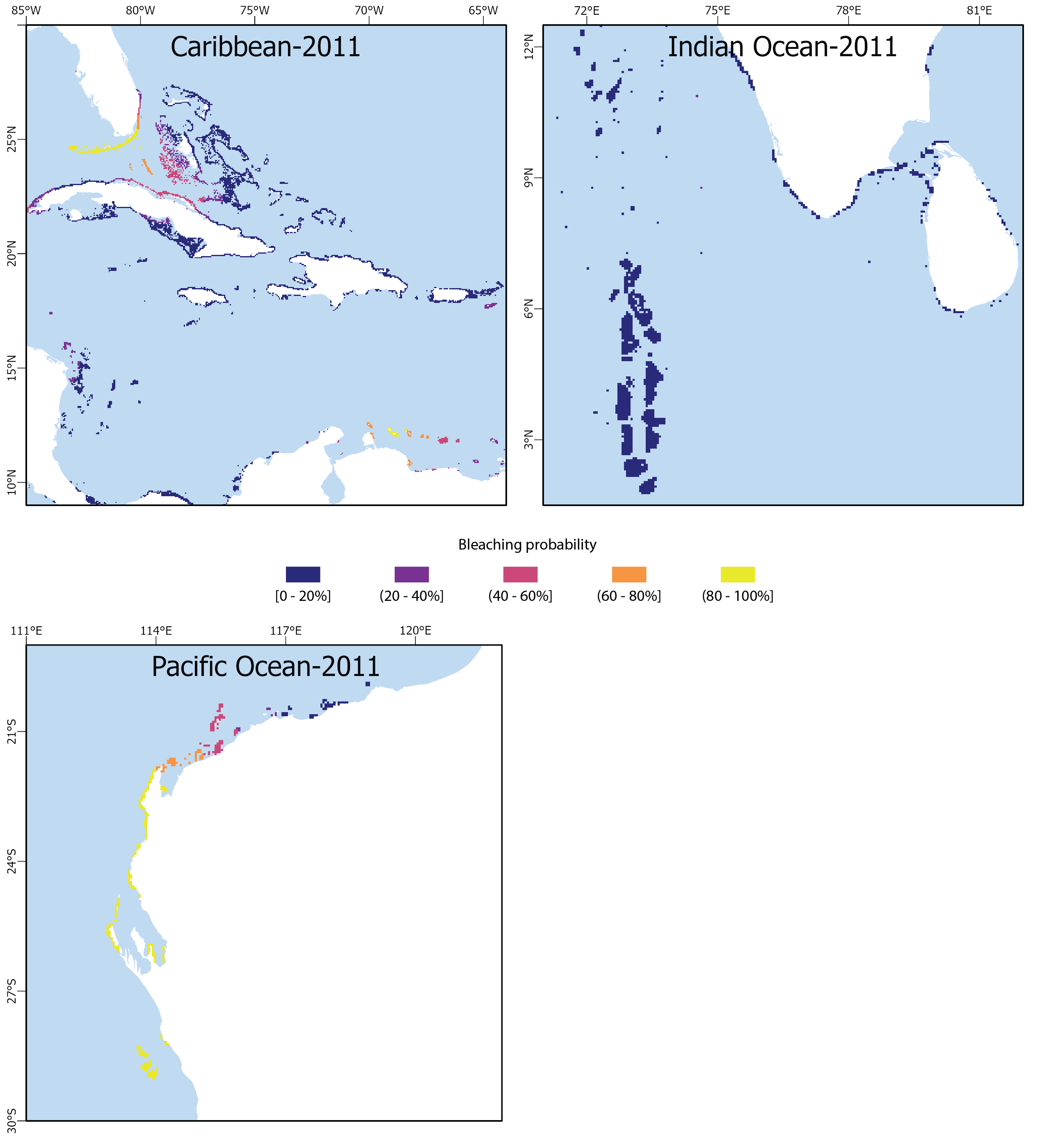


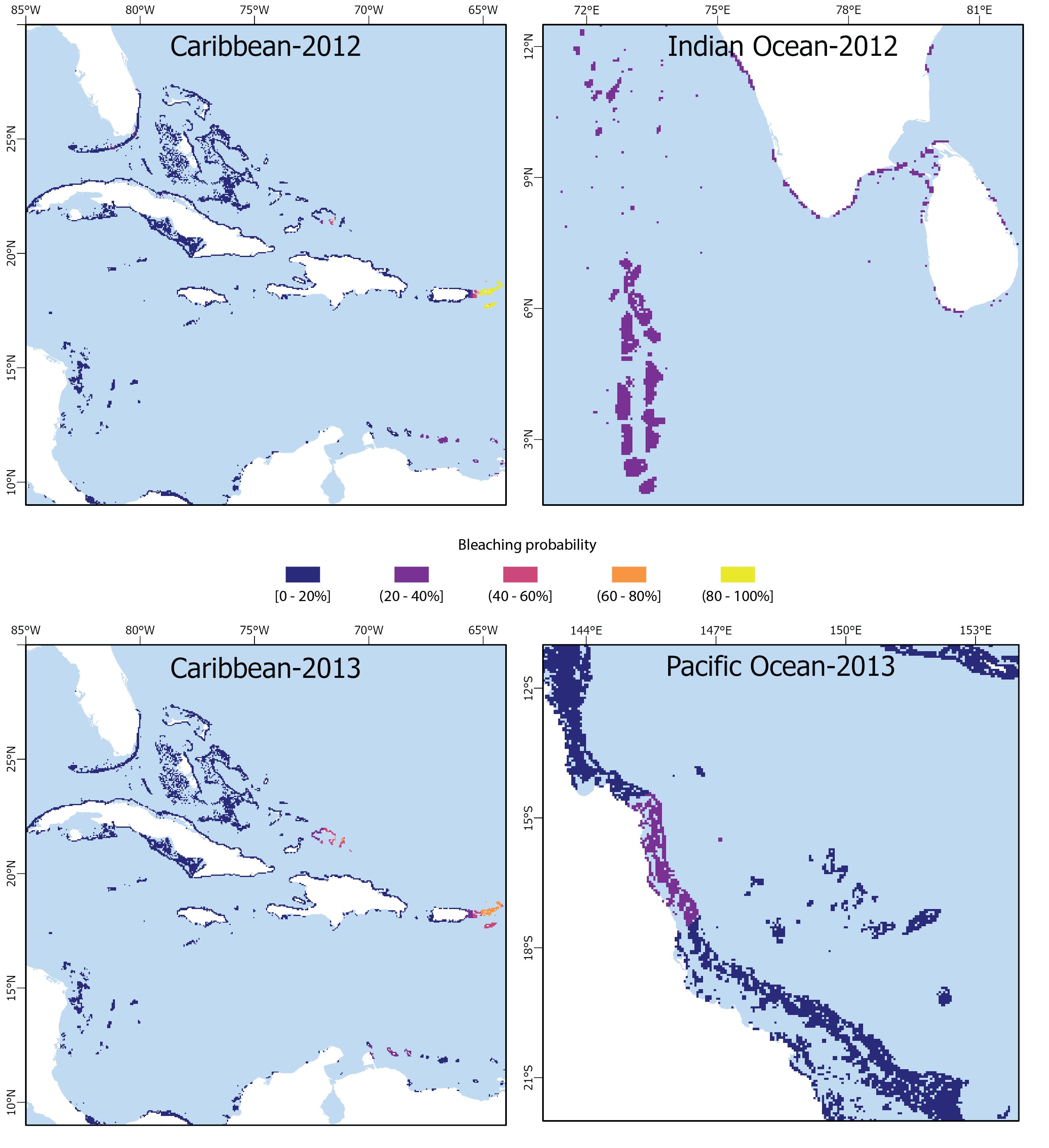


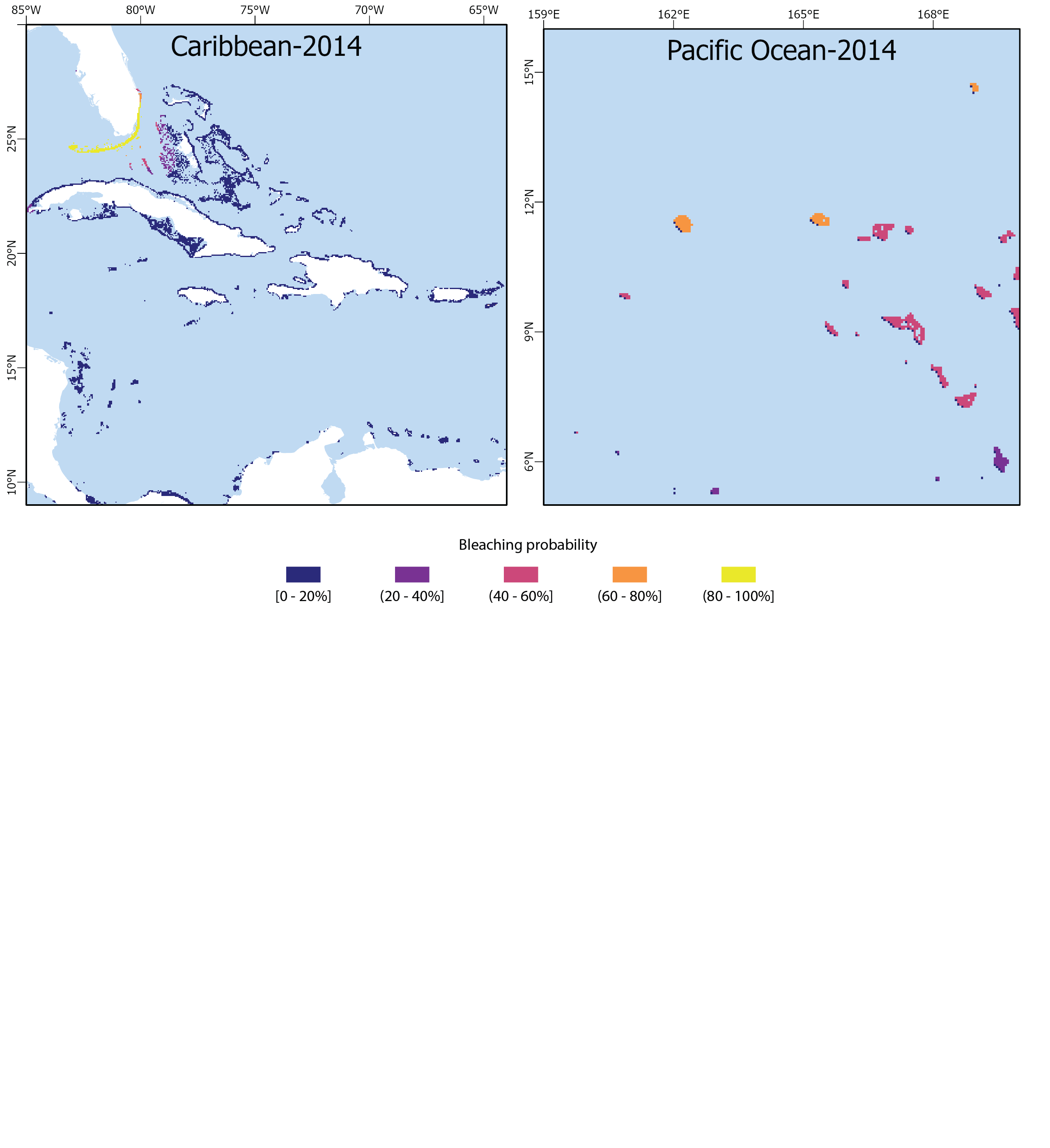


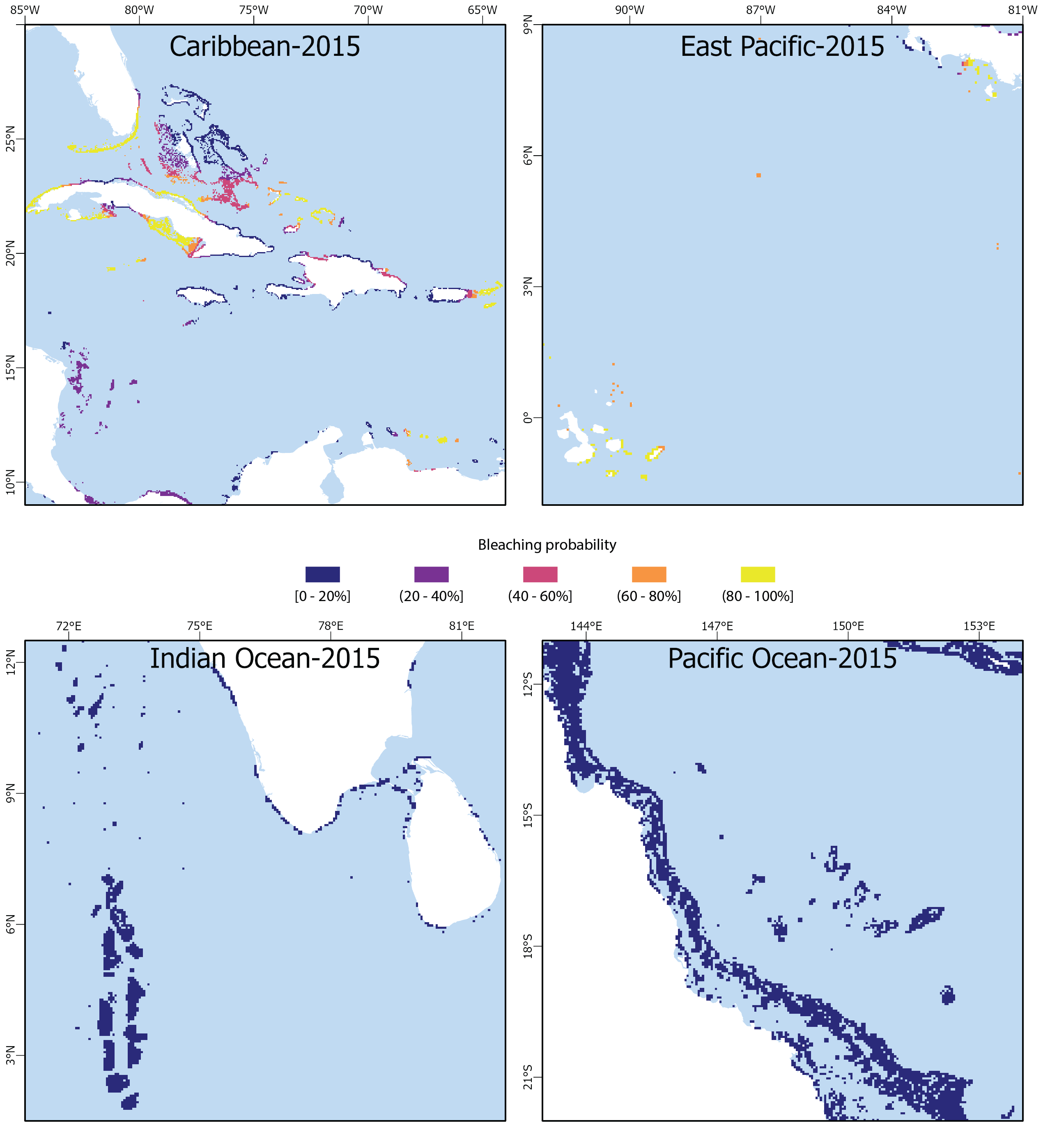


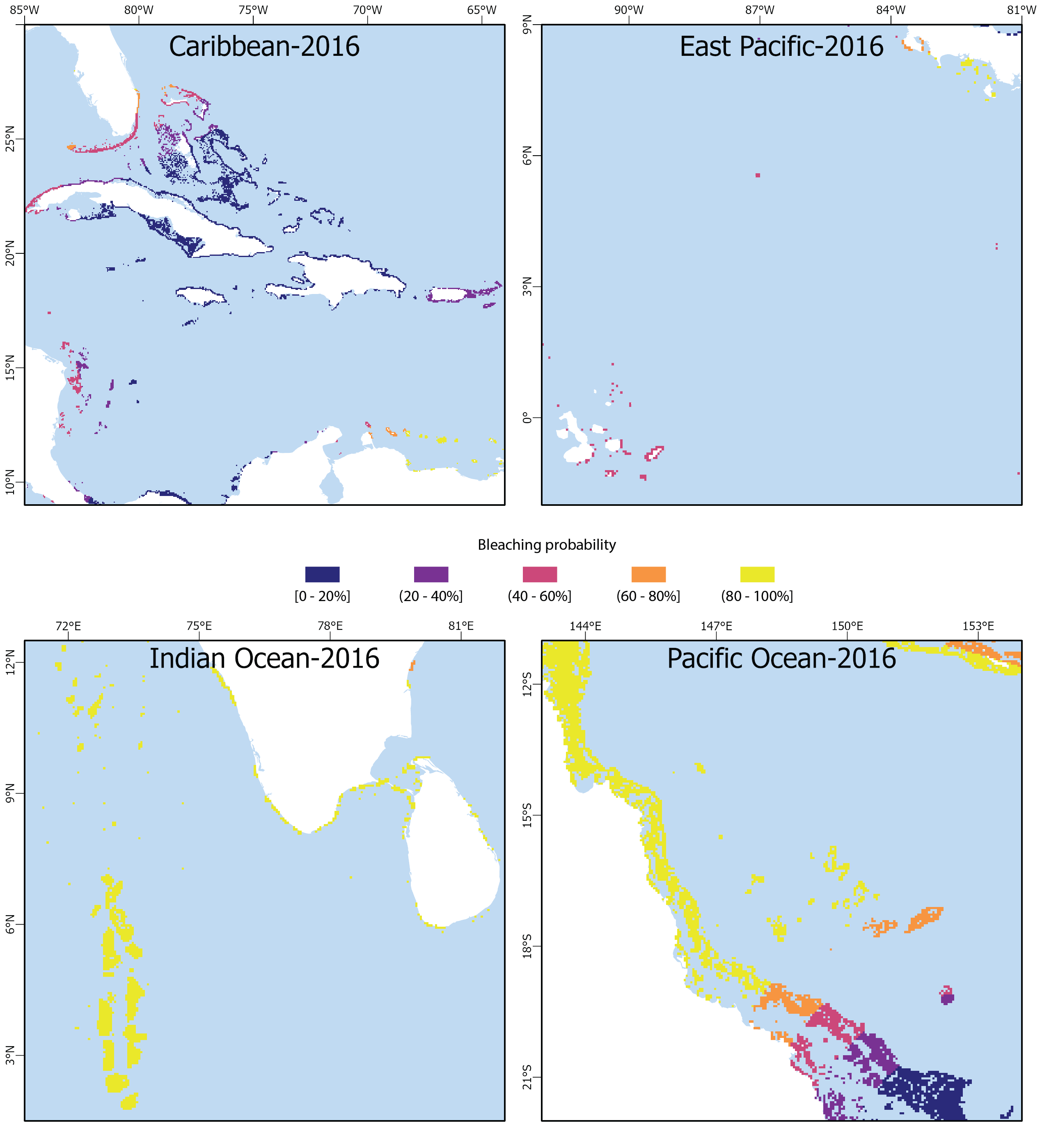


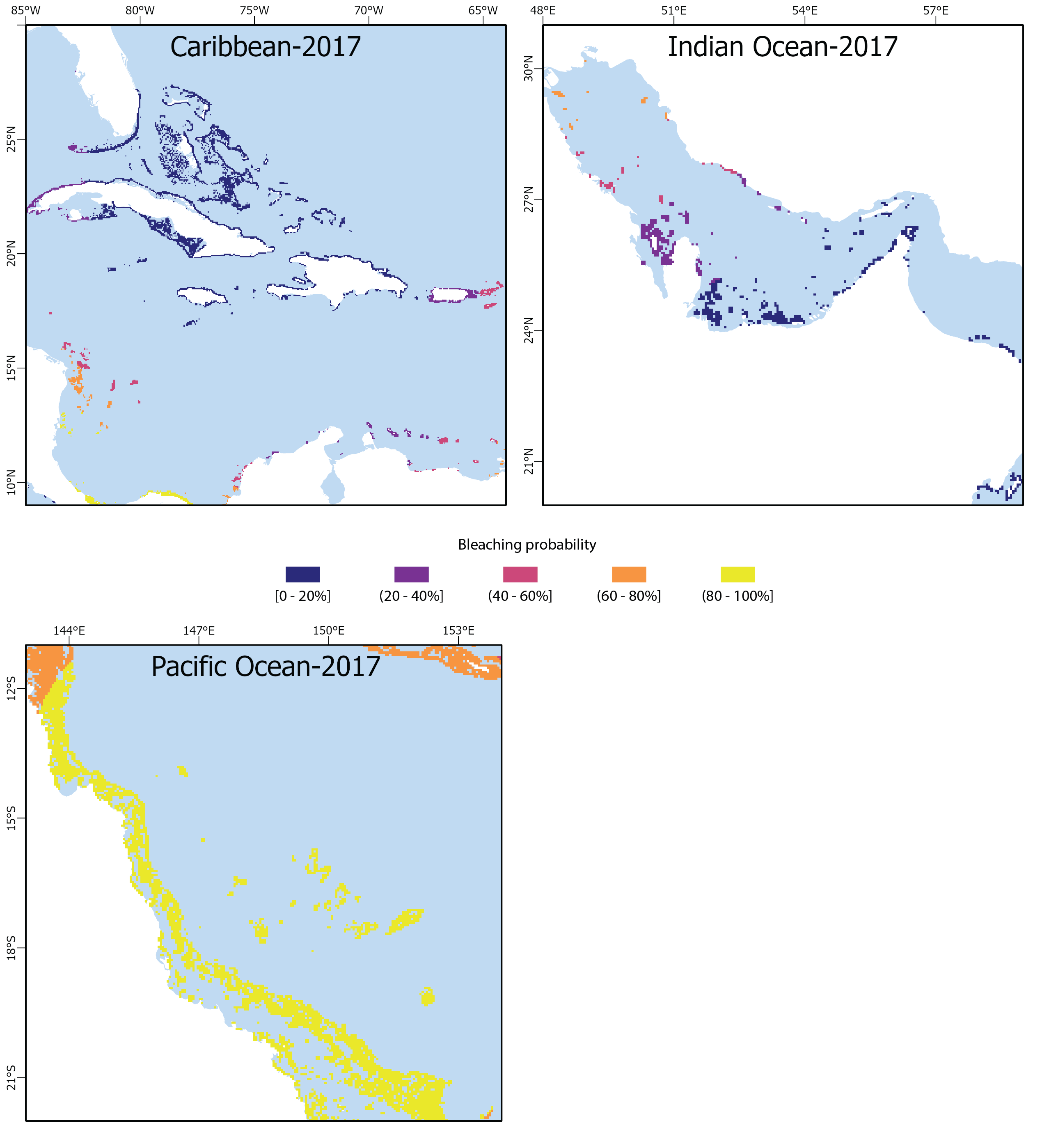

Supplement: S6 Fig — (DOCX) [file pone.0281719.s006.docx]
