# Supplementary material for: Increase in the extent of mass coral bleaching over the past half-century, based on an updated global database
Source: PLoS One. 2023 Feb 13;18(2):e0281719. doi: 10.1371/journal.pone.0281719 (PMC9925063; doi:10.1371/journal.pone.0281719)
Supplement: S1 Table — (DOCX) [file pone.0281719.s007.docx]

**S1 Table. Coral bleaching observational database legend**

| Category | Description |
| --- | --- |
| **Ocean_Region** | See S2 Figure |
| **Country** | Follows ReefBase convention |
| **Location** | State, region or island |
| **Site_Name** | Dive site or local community |
| **Latitude** | In decimal degrees |
| **Longitude** | In decimal degrees |
| **Date** | Date of observation * |
| **Month** | Month of observation * |
| **Year** | Year of observation |
| **Depth** | Depth of observation * |
| **Severity_Code** | Categorical variable, following Reefbase protocol |
| **Percent_Bleached** | Percent of coral cover bleached |
| **Mortality Code** | Categorical variable, following Reefbase protocol |
| **Percent_Mortality** | Percent mortality, as a percentage of coral cover * |
| **Survey_Type** | Survey type (e.g., random dive, point intercept transects) * |
| **Source** | Initial source of the report (i.e., existing database or research group) |
| **Citation** | Source manuscript or report |
| **Comments** | Other comments on the report |
| **Database_Code** | 1 = ReefBase reports in V1, 2 = V1 additions, 3 = V2 additions from ReefBase,  4 = V2 additions from literature search and outreach, 5 = V2 additions from Coral Reef Watch 2014-2017 database |
|  |  |

*if available
