# Supplementary material for: Increase in the extent of mass coral bleaching over the past half-century, based on an updated global database
Source: PLoS One. 2023 Feb 13;18(2):e0281719. doi: 10.1371/journal.pone.0281719 (PMC9925063; doi:10.1371/journal.pone.0281719)
Supplement: S2 Table — Summary statistics of bleaching probabilities for cells with reports are shown. (DOCX) [file pone.0281719.s008.docx]

**S2 Table. Comparison of different thresholds to define pseudo-absences**. Summary statistics of bleaching probabilities for cells with reports are shown.

| **Region** | **Year** | **Pseudo-absence threshold** | **Mean** | **Median** | **SD** |
| --- | --- | --- | --- | --- | --- |
| **Caribbean** | 1987 | DHW = 0 | 0.99881 | 1 | 0.00422 |
| **Caribbean** | 1987 | DHW <0.25 | 0.67358 | 0.88996 | 0.39167 |
| **Caribbean** | 1987 | DHW <0.5 | 0.55484 | 0.70117 | 0.37638 |
| **Caribbean** | 1987 | DHW <1 | 0.28374 | 0.12477 | 0.31806 |
| **Caribbean** | 2005 | DHW = 0 | 0.99629 | 1 | 0.03054 |
| **Caribbean** | 2005 | DHW <0.25 | 0.98852 | 0.99988 | 0.06909 |
| **Caribbean** | 2005 | DHW <0.5 | 0.97701 | 0.99928 | 0.10574 |
| **Caribbean** | 2005 | DHW <1 | 0.97199 | 0.99961 | 0.12549 |
| **East Pacific** | 1998 | DHW = 0 | - | - | - |
| **East Pacific** | 1998 | DHW <0.25 | 0.97472 | 0.99440 | 0.05946 |
| **East Pacific** | 1998 | DHW <0.5 | 0.90810 | 0.94683 | 0.10772 |
| **East Pacific** | 1998 | DHW <1 | - | - | - |
| **East Pacific** | 2015 | DHW = 0 | - | - | - |
| **East Pacific** | 2015 | DHW <0.25 | - | - | - |
| **East Pacific** | 2015 | DHW <0.5 | 0.99998 | 1 | 0.00005 |
| **East Pacific** | 2015 | DHW <1 | 0.99980 | 1 | 0.00052 |
| **Indian Ocean** | 2010 | DHW = 0 | 0.92810 | 0.99939 | 0.19414 |
| **Indian Ocean** | 2010 | DHW <0.25 | 0.85671 | 0.97672 | 0.27017 |
| **Indian Ocean** | 2010 | DHW <0.5 | 0.90217 | 0.99244 | 0.20827 |
| **Indian Ocean** | 2010 | DHW <1 | 0.68860 | 0.80507 | 0.33811 |
| **Indian Ocean** | 2016 | DHW = 0 | - | - | - |
| **Indian Ocean** | 2016 | DHW <0.25 | 0.95391 | 0.98963 | 0.08286 |
| **Indian Ocean** | 2016 | DHW <0.5 | 0.92103 | 0.98640 | 0.12274 |
| **Indian Ocean** | 2016 | DHW <1 | 0.89919 | 0.96672 | 0.13808 |
| **Pacific Ocean** | 2000 | DHW = 0 | 0.95333 | 1 | 0.10071 |
| **Pacific Ocean** | 2000 | DHW <0.25 | 0.90126 | 0.96267 | 0.15615 |
| **Pacific Ocean** | 2000 | DHW <0.5 | 0.71261 | 0.85159 | 0.31190 |
| **Pacific Ocean** | 2000 | DHW <1 | 0.39615 | 0.45508 | 0.16791 |
| **Pacific Ocean** | 2016 | DHW = 0 | 0.93355 | 0.99734 | 0.17812 |
| **Pacific Ocean** | 2016 | DHW <0.25 | 0.90941 | 0.99079 | 0.21369 |
| **Pacific Ocean** | 2016 | DHW <0.5 | 0.87984 | 0.98682 | 0.24816 |
| **Pacific Ocean** | 2016 | DHW <1 | 0.82764 | 0.97224 | 0.27989 |
| - No result because semivariograms failed to converge | | | | | |
