# Supplementary material for: Increase in the extent of mass coral bleaching over the past half-century, based on an updated global database
Source: PLoS One. 2023 Feb 13;18(2):e0281719. doi: 10.1371/journal.pone.0281719 (PMC9925063; doi:10.1371/journal.pone.0281719)
Supplement: S3 Table — (DOCX) [file pone.0281719.s009.docx]

S3 Table. Years and regions for which indicator kriging could be conducted

| **Year** | **Caribbean** | **East Pacific** | **Indian Ocean** | **Pacific Ocean** |
| --- | --- | --- | --- | --- |
| **1985** |  |  |  |  |
| **1986** |  |  |  |  |
| **1987** | ✓ |  |  |  |
| **1988** | ✓ |  |  |  |
| **1989** |  |  |  |  |
| **1990** |  |  |  |  |
| **1991** |  |  |  | ✓ |
| **1992** |  |  |  |  |
| **1993** |  |  |  |  |
| **1994** |  |  |  | ✓ |
| **1995** | ✓ |  |  |  |
| **1996** |  |  |  | ✓ |
| **1997** |  | ✓ |  | ✓ |
| **1998** | ✓ | ✓ | ✓ | ✓* |
| **1999** | ✓ |  |  |  |
| **2000** | ✓ |  | ✓ | ✓ |
| **2001** | ✓ |  | ✓ | ✓ |
| **2002** | ✓ |  | ✓ | ✓* |
| **2003** | ✓ |  | ✓ |  |
| **2004** | ✓* |  |  | ✓* |
| **2005** | ✓ |  | ✓ |  |
| **2006** | ✓* |  |  |  |
| **2007** | ✓ |  | ✓ | ✓ |
| **2008** | ✓* |  |  | ✓* |
| **2009** | ✓ |  | ✓ | ✓ |
| **2010** | ✓ |  | ✓ | ✓ |
| **2011** | ✓ |  | ✓ | ✓ |
| **2012** | ✓* |  | ✓* |  |
| **2013** | ✓ |  |  | ✓ |
| **2014** | ✓ |  |  | ✓* |
| **2015** | ✓ | ✓ | ✓ | ✓ |
| **2016** | ✓ | ✓* | ✓* | ✓ |
| **2017** | ✓* |  | ✓* | ✓ |
| * Semivariogram completed by providing an initial estimate for range, sill, and nugget values | | | | |
