# Supplementary material for: Increase in the extent of mass coral bleaching over the past half-century, based on an updated global database
Source: PLoS One. 2023 Feb 13;18(2):e0281719. doi: 10.1371/journal.pone.0281719 (PMC9925063; doi:10.1371/journal.pone.0281719)
Supplement: S4 Table — Results of Welch two-sample t-tests between database Version 1 (V1) and Version 2 (V2, this study) for each year and region where indicator kriging was conducted in both versions. (DOCX) [file pone.0281719.s010.docx]

**S4 Table. Database comparison.** Results of Welch two-sample t-tests between database Version 1 (V1) and Version 2 (V2, this study) for each year and region where indicator kriging was conducted in both versions

| **Year** | **Region** | **Mean V1** | **Mean V2** | **t-stat** | **p-value** |
| --- | --- | --- | --- | --- | --- |
| 1987 | Caribbean | 0.6498852 | 0.5549954 | 0.8072611 | 0.4289981 |
| 1988 | Caribbean | 0.489886 | 0.0692227 | 1.9495456 | 0.1070474 |
| 1995 | Caribbean | 0.009717 | 0.4525482 | -6.3625027 | 0.0000813* |
| 1997 | East Pacific | 0.8594734 | 0.9122989 | -1.1009108 | 0.2758041 |
| 1998 | Caribbean | 0.0000052 | 0.692704 | -23.331146 | 0* |
| 1998 | East Pacific | 0.7112802 | 0.9088689 | -8.1229562 | 0* |
| 1998 | Indian Ocean | 0.4650539 | 0.8820459 | -14.963371 | 0* |
| 1998 | Pacific Ocean | 0.322683 | 0.7064251 | -23.68172 | 0* |
| 1999 | Caribbean | 0.6296439 | 0.3466263 | 3.6086274 | 0.0017222* |
| 2000 | Pacific Ocean | 0.4821943 | 0.6531391 | -3.5659993 | 0.0004817* |
| 2001 | Pacific Ocean | 0.8168254 | 0.0640222 | 14.1498585 | 0* |
| 2002 | Caribbean | 0.2417538 | 0.0660955 | 7.7176127 | 0.0002031* |
| 2002 | Indian Ocean | 0.2245842 | 0.2788097 | -0.7426841 | 0.4659617 |
| 2002 | Pacific Ocean | 0.0000904 | 0.7032597 | -41.00425 | 0* |
| 2003 | Caribbean | 0.0894908 | 0.1662759 | -1.2163401 | 0.2463958 |
| 2004 | Caribbean | 0.6666485 | 0.2836232 | 5.8389517 | 0.0000003* |
| 2004 | Pacific Ocean | 0.8648563 | 0.2199339 | 8.0320718 | 0* |
| 2005 | Caribbean | 0.890003 | 0.9770231 | -19.719136 | 0* |
| 2005 | Indian Ocean | 0.5477842 | 0.7952154 | -4.7181035 | 0.0000092* |
| 2006 | Caribbean | 0.7562746 | 0.7070138 | 3.2796662 | 0.0010813* |
| 2007 | Caribbean | 0.0691534 | 0.506591 | -17.418555 | 0* |
| 2007 | Indian Ocean | 0.2565017 | 0.3776686 | -1.3629354 | 0.1845871 |
| 2007 | Pacific Ocean | 0.6204642 | 0.2292471 | 4.5915236 | 0.0000813* |
| 2008 | Caribbean | 0.8059882 | 0.2069577 | 7.3562717 | 0.0002405* |
| 2008 | Pacific Ocean | 0.0117727 | 0.0614809 | -4.0915975 | 0.0003056* |
| 2009 | Caribbean | 0.4210708 | 0.4606641 | -1.0128609 | 0.3193843 |
| 2009 | Pacific Ocean | 0.0591297 | 0.1030499 | -1.9341716 | 0.0589201 |
| 2010 | Indian Ocean | 0.6752651 | 0.9167018 | -7.1590388 | 0* |
| 2010 | Pacific Ocean | 0.1435075 | 0.8782891 | -32.568945 | 0* |

* Significantly different (p < 0.05)
