# Supplementary material for: Increase in the extent of mass coral bleaching over the past half-century, based on an updated global database
Source: PLoS One. 2023 Feb 13;18(2):e0281719. doi: 10.1371/journal.pone.0281719 (PMC9925063; doi:10.1371/journal.pone.0281719)
Supplement: S5 Table — Results are shown for cells with different ranges of bleaching probability. (DOCX) [file pone.0281719.s011.docx]

**S5 Table. Thermal stress for reef cells, by region**. Results are shown for cells with different ranges of bleaching probability.

| **Australia** | | | | | | | |
| --- | --- | --- | --- | --- | --- | --- | --- |
| **Mean Annual maximum DHW (°C-weeks)** | **All cells** | **>90%** | **>66–90%** | **>50–66%** | **>33–50%** | **>10–33%** | **≤10** |
| **All years** | 2.09* | 6.66* | 5.19 | 5.17 | 4.75* | 2.97* | 1.49* |
| **1997-1998** | 0.91* | 3.82* | 2.29 | 1.94 | 2.19 | 1.61* | 0.5* |
| **2009-2010** | 2.88* | - | 4.8 | 4.94 | 7.17 | 4.8 | 2.14* |
| **2014-2016** | 2.66* | 7.57* | 6.7* | 4.74* | 3.83* | 1.7* | 1.22* |
| **Caribbean** | | | | | | | |
| **Mean Annual maximum DHW (°C-weeks)** | **All cells** | **>90%** | **>66–90%** | **>50–66%** | **>33–50%** | **>10–33%** | **≤10** |
| **All years** | 1.98* | 5.6* | 5.23* | 3.9* | 3.73* | 2.82* | 0.94* |
| **1997-1998** | 2.98* | 4.91* | 4.53* | 4.05* | 2.98* | 2.68* | 1.09* |
| **2009-2010** | 3.3* | 5.73* | 7.21 | 4.97 | 5.38 | 2.67* | 1.46* |
| **2014-2016** | 2.38* | 5.2* | 3.58* | 3.09* | 3.85* | 2.68* | 0.99* |
| **South East Asia** | | | | | | | |
| **Mean Annual maximum DHW (°C-weeks)** | **All cells** | **>90%** | **>66–90%** | **>50–66%** | **>33–50%** | **>10–33%** | **≤10** |
| **All years** | 1.28* | 6.46* | 5.49* | 4.12* | 3.96* | 3.69* | 0.88* |
| **1997-1998** | 1.23* | 5.73* | 4.12* | 3.06 | 3.22 | 2.38* | 0.52* |
| **2009-2010** | 2.43* | 6.2* | 4.96* | 4.13* | 3.63 | 3.75 | 1.15* |
| **2014-2016** | 2.47* | 7.33* | 7.02* | 4.74* | 4.31* | 4.09* | 1.49* |
| **W Indian** | | | | | | | |
| **Mean Annual maximum DHW (°C-weeks)** | **All cells** | **>90%** | **>66–90%** | **>50–66%** | **>33–50%** | **>10–33%** | **≤10** |
| **All years** | 1.42* | 5.44* | 4.21* | 3.05* | 2.47* | 1.12* | 0.66* |
| **1997-1998** | 3.28* | 4.27* | 3.67* | 2.48* | 1.77* | 1.49* | 0.63* |
| **2009-2010** | 2.1* | 3.97 | 4.12 | 3 | 3.44 | 3.15 | 1.56* |
| **2014-2016** | 3.19 | 6.35* | 4.98* | 4.14 | 3.3 | 1.18* | 0.47* |
| **Polynesia** | | | | | | | |
| **Mean Annual maximum DHW (°C-weeks)** | **All cells** | **>90%** | **>66–90%** | **>50–66%** | **>33–50%** | **>10–33%** | **≤10** |
| **All years** | 1.08* | 6.69* | 5.24* | 3.88* | 3.08 | 3.24 | 0.58* |
| **1997-1998** | 0.46* | - | 3.39 | 3.18 | 2.12* | 1.52* | 1.52* |
| **2009-2010** | 0.77* | 3.18 | 3.71 | 3.67 | 1.28 | 1.26 | 0.69* |
| **2014-2016** | 3.01* | 6.68* | 4.99 | 4.53 | 4.76 | 4.08* | 1.66* |
| **C Indian** | | | | | | | |
| **Mean Annual maximum DHW (°C-weeks)** | **All cells** | **>90%** | **>66–90%** | **>50–66%** | **>33–50%** | **>10–33%** | **≤10** |
| **All years** | 1.49* | 6.44* | 4.05* | 3.67* | 2.42* | 1.03* | 0.6* |
| **1997-1998** | 3.39 | 4.82* | 3.31 | 2.6* | 1.44 | 1.36 | 1.18 |
| **2009-2010** | 1.94* | 6.8* | 4.26* | 2.21* | 2.18* | 3.05* | 0.72* |
| **2014-2016** | 4.58* | 7.86* | 5.26* | 5.64* | 3.63 | 3.19 | 1.77* |
| **Micronesia** | | | | | | | |
| **Mean Annual maximum DHW (°C-weeks)** | **All cells** | **>90%** | **>66–90%** | **>50–66%** | **>33–50%** | **>10–33%** | **≤10** |
| **All years** | 1.76* | 8.60* | 9.71* | 5.73 | 4.11* | 5.60 | 1.02* |
| **1997-1998** | 0.74* | 5.59* | 4.09* | 1.49* | 12.66* | 1.98* | 0.42* |
| **2009-2010** | 2.03* | 7.31* | 4.71 | 3.23* | 4.18 | 8.38 | 1.52* |
| **2014-2016** | 4.55* | 10.41* | 10.73* | 5.80* | 3.04 | 4.52 | 2.43* |
| **Melanesia** | | | | | | | |
| **Mean Annual maximum DHW (°C-weeks)** | **All cells** | **>90%** | **>66–90%** | **>50–66%** | **>33–50%** | **>10–33%** | **≤10** |
| **All years** | 1.73* | 7.69* | 6.08* | 4.43 | 4.24 | 3.79* | 1.40* |
| **1997-1998** | 0.50* | - | 6.29* | - | - | 1.54* | 0.47* |
| **2009-2010** | 1.73* | - | - | 2.59 | 2.71 | 2.75 | 1.56* |
| **2014-2016** | 3.86* | 7.97* | 7.50* | 5.27 | 6.59 | 4.96* | 2.58* |
| **Middle East** | | | | | | | |
| **Mean Annual maximum DHW (°C-weeks)** | **All cells** | **>90%** | **>66–90%** | **>50–66%** | **>33–50%** | **>10–33%** | **≤10** |
| **All years** | 1.92* | 7.04* | 5.32 | 5.69 | 6.03 | 2.90* | 1.25* |
| **1997-1998** | 3.55* | 8.09* | 4.17 | 4.82 | 3.85* | 2.95* | 1.82* |
| **2009-2010** | 2.55* | 7.05* | 5.14 | 5.92 | 6.81 | 4.26* | 1.20* |
| **2014-2016** | 3.65* | 5.90 | 5.65 | 6.82 | 7.16 | 6.15 | 2.45* |
| **East Pacific** | | | | | | | |
| **Mean Annual maximum DHW (°C-weeks)** | **All cells** | **>90%** | **>66–90%** | **>50–66%** | **>33–50%** | **>10–33%** | **≤10** |
| **All years** | 7.78 | 13.31* | 8.11 | 5.47 | 5.39 | 2.09* | 0.69* |
| **1997-1998** | 13.70* | 22.93* | 7.34* | 0.41 | 0.13 | 0.00 | 0.00 |
| **2009-2010** | 0.12 | - | - | - | - | - | 0.12 |
| **2014-2016** | 5.06 | 4.78 | 8.94 | 6.92 | 5.56 | 2.51* | 0.66* |
| *Statistically significant (p <0.05) from other bleaching probability groups for that time period, with higher probability group featuring higher mean DHW | | | | | | | |
